# ScRNA-seq reveals dynamic macrophage heterogeneity in chronic liver disease progression and prognostic biomarkers *KLF2/SPP1* in HCC

**DOI:** 10.3389/fimmu.2026.1766301

**Published:** 2026-02-18

**Authors:** Qi Pan, Xinru Wang, Borui Li, Zhenzhen Cai, Shuwen Chen, Jiahong Hu, Xuenan Yuan, Jie Yang, An-Yuan Guo, Zhihong Zhang

**Affiliations:** 1 School of Life and Health Sciences, Institute of Biomedical Research, Hainan Province Key Laboratory of One Health, Collaborative Innovation Center of Life and Health, Hainan University, Haikou, Hainan, China; 2 State Key Laboratory of Digital Medical Engineering, Britton Chance Center for Biomedical Photonics, School of Biomedical Engineering, Hainan University, Sanya, Hainan, China; 3 MOE Key Laboratory for Biomedical Photonics, Wuhan National Laboratory for Optoelectronics-Huazhong University of Science and Technology, Wuhan, Hubei, China; 4 Department of Thoracic Surgery, West China Biomedical Big Data Center, West China Hospital, Sichuan University, Chengdu, China

**Keywords:** cell heterogeneity, chronic liver diseases processes, macrophage-T cell interactions, prognostic biomarkers, scRNA-seq

## Abstract

**Background:**

Metabolic dysfunction-associated steatohepatitis (MASH)-induced chronic liver diseases (CLDs) were worldwide prevalence and incidence. The stage-resolved cellular and molecular programs remained incompletely defined. This study aimed to resolve stage-specific immune and transcriptional features across CLDs processes and to identify prognostic biomarkers.

**Methods:**

We integrated single-cell RNA sequencing datasets from healthy liver, MASH, cirrhosis and HCC to construct a stage-resolved cellular atlas. We performed cell-state scoring, diffusion pseudotime, gene regulatory network inference, and cell–cell interaction to decipher various macrophages and T cells transcriptional profiles. We established a method of gene sets enrichment score to detect prognostic markers and employed RNA fluorescence *in situ* hybridization (FISH) to validate macrophage subtype abundances and spatial interactions.

**Results:**

The integrated atlas revealed the heterogeneity cell-subtype composition and transcriptional features across CLD stages. In MASH, CXCL3^+^ macrophage and CXCL10^+^ macrophage were enriched and characterized by *ETS2*- and *IRF1*-driven inflammatory programs that might potentially contribute to the transition from MASH to HCC. SPP1^+^ macrophage was exclusive to HCC and might contribute to cytotoxic T-cell (Tc) dysfunction but do not directly demonstrate functional suppression or exhaustion.

Subsequently, we sought to validate the robustness of these signature genes. We integrated clinical datasets from the TCGA-LIHC to validate signature genes in HCC derived from the scRNA-seq results and identify prognostic biomarker. Survival-linked analyses uncovered SPP1 and KLF2 as prognostic biomarkers. FISH confirmed stage-specific shifts in macrophage abundances and close spatial interactions between SPP1^+^ macrophages and Tc in HCC specimens.

**Conclusion:**

We provided a stage-resolved framework to delineated macrophage heterogeneity during CLDs progression and identified SPP1 and KLF2 as candidate prognostic biomarkers and potential therapeutic targets in HCC.

## 1 Introduction

Chronic liver diseases (CLDs) presented a significant global health burden, with a mortality rate of two million deaths per year (1). CLD was also a continuous and progressive deterioration of liver functions (2). The distribution of CLDs was changing, metabolic dysfunction–associated steatohepatitis (MASH) has become the most prevalent cause of CLD, and its global incidence and prevalence were steadily rising within the next decade significantly (3). Cirrhosis was a leading cause of death among patients of CLD worldwide, frequently progressed to hepatocellular carcinoma (HCC) (4). Although the rapidly evolving single-cell RNA sequencing (scRNA-seq) technologies have provided valuable insight into the pathogenesis of liver diseases (5, 6), comprehensive analysis of diverse cellular populations and phenotypic states during the transition from MASH to advanced liver disease and HCC remained limited. Hence, a deep understanding of the stage-specific immune landscapes and transcriptional programs of CLDs was urgently needed to improve the rational design of immunotherapies.

The immune system played a critical role in CLDs (7). It has been reported that both innate and adaptive immune cells shaped the hepatic microenvironment during the progression from MASH to fibrosis, cirrhosis, and HCC (8). Macrophages (Macs) represent a first-line defense in the liver, they showed impaired turnover in MASH and could be driven toward pro-inflammatory activation by the excess lipids and tissue damage in the fatty liver (9). The scar-associated macrophages (SAMacs), displaying a pro-fibrogenic phenotype, have been identified expand in liver cirrhosis (5). Tumor-associated macrophages (TAMs) exhibited distinct transcriptional states and were associated with poor prognosis in HCC (10). However, the cellular and molecular mechanisms involved in CLDs pathogenesis remain largely unknown.

Here, we integrated 29 samples of scRNA-seq data from healthy liver, MASH, cirrhosis and HCC to identify stage-specific macrophage and T cell subpopulations across chronic liver disease processes. We clarified the molecular dynamics of C1QA^+^ Mac, CXCL3^+^ Mac, CXCL10^+^ Mac, SAMac and SPP1^+^ Mac along CLDs processes, and discovered the transcription factor *ETS2* (ETS proto-oncogene 2) and *IRF1* (interferon regulatory factor 1) as key regulators in macrophages, potentially driving the transitions of MASH to HCC. Moreover, we revealed SPP1^+^ macrophages established anergic interactions with cytotoxic T cell (Tc), with *SPP1* serving as a poor prognostic biomarker, whereas *KLF2* (KLF transcription factor 2) exhibited an anti-tumor phenotype in HCC.

## 2 Materials and methods

### 2.1 Data collection

ScRNA-seq data were collected from Gene Expression Omnibus (GEO; https://www.ncbi.nlm.nih.gov/geo/) database and Mendeley Data (https://data.mendeley.com/). The inclusion criteria for scRNA-seq datasets were as follows: (1) liver tissue samples with clearly annotated disease status relevant to MASH, liver cirrhosis, or HCC; (2) the availability of raw or processed gene expression matrices and corresponding cell-type annotations; (3) the scRNA-seq analysis results have been confirmed by FISH, flow cytometry, immunostaining, or experimental studies in original article. We selected 5 cases of healthy controls and 5 cases of liver cirrhotic patients from GSE136103, (5) 3 cases of MASH patients from GSE159977. (6) Because the annual incidence of hepatocellular carcinoma arising from NASH-related cirrhosis was relatively low, we were unable to collect the dataset. Viral hepatitis-induced and NASH-induced HCC have been reported to share overlapping inflammatory and immune signaling pathways that drive malignant transformation (11). Therefore, we collected 6 cases of HBV-related cirrhosis with HCC from skrx2fz79n (12) as a surrogate. The data accession numbers and information were provided in **Supplementary Table 1**. Additionally, the RNA expression data and clinical information of HCC used in this study were obtained from The Cancer Genome Atlas public access web portal (TCGA-LIHC; https://portal.gdc.cancer.gov/).

### 2.2 Single-cell RNA-seq data analysis

Cases from GSE136103 and GSE159977 were obtained raw fastq files. They were processed with the Cell Ranger pipeline (version 6.0.2, 10X Genomics) and mapped to GRCh38 reference genome to generate count matrices. The count matrix of skrx2fz79n dataset was obtained from rds files (https://data.mendeley.com/datasets/skrx2fz79n/1). We used the “emptyDrops” function of the R package DropletUtils (version 1.14.2) (13) to remove barcode-swapped pseudo-cells. We applied “doubletFinder_v3” function of R package DoubletFinder (version 2.0.3) (14) to identify doublets. For quality control, cells with mitochondrial gene percentages less than 10%, genes expressed in over 3 cells, and detected genes between 200 to 4000 were kept. The R package Seurat (version 4.2.1) (15) was utilized to perform data normalization and dimensionality reduction. Gene expression counts were normalized and scaled by “SCTransform” function with glmGamPoi model, and we calculated a PCA matrix by “RunPCA” function. After PCA, we used the “RunHarmony” function with SCT assay for batch effect correction and datasets integration in the R package harmony (version 0.1.0) (16).

### 2.3 Unsupervised clustering and cell-type annotation

The top 40 harmony dimensions were used to carry out the uniform manifold approximation and projection (UMAP) dimensional reduction. We then constructed the nearest-neighbour graph by the “FindNeighbors” function with the reduction as ‘harmony’. The “FindClusters” function was then used to identify clusters with the resolution parameter of 0.09 of the whole object, which resulted in 15 clusters; the resolution of 0.3 for T cells and 0.4 for mononuclear phagocytes. To identify cluster-specific marker genes, we used “FindAllMarkers” function to select those detect in a minimum of 25% of cells within the cluster, displaying a *p value* < 0.05 in the Wilcoxon rank-sum test, and demonstrating a differential expression threshold of 0.25 log fold change (log2FC). The main cell types were annotated with known cell-type marker genes based on CellTypist (https://www.celltypist.org/) (17) and CellMarker 2.0 (http://117.50.127.228/CellMarker/) (18). We applied type-specific markers to annotate mononuclear phagocyte and T cell subtypes.

### 2.4 Trajectory inference and cell-cell communication

Trajectory and diffusion-pseudotime (DPT) analysis were generated using the R package destiny (version 3.22.0) (19). The number of nearest neighbors, k, was set to 10. The principal components were set to 30. The R package CellChat (version 1.6.1) (20) was utilized to explore the communication of macrophages and T cells into functionally relevant signaling pathways. We used the processed expression matrices from the “SCT” data slot of the Seurat object with corresponding annotations to create a CellChat object. CellChatDB.human was set as the ligand-receptor interaction database. We applied the minimum cell count criterion of 10.

### 2.5 Macrophage and T cell states

We used the R package AUCell (21) to calculate the signature score of curated gene sets relate to macrophage and T cell functional states (22, 23) (**Supplementary Tables 2, 3**). AUCell could provide a relative measure of gene importance in each cell to evaluate the degree of gene set enrichment. The ranked gene expression matrix was built by the AUCell_buildRankings function, and then we calculated the AUC value using the AUCell_calcAUC function.

### 2.6 Functional enrichment analysis and SCENIC analysis

We performed GO enrichment analysis with the R package clusterProfiler (version 4.2.2) (24). The top 8 GO annotations were chosen for C1QA^+^ Mac and SPP1^+^ Mac for visualization. Enrichment scores for the eight selected GO annotations were calculated by a hypergeometric statistical test with a significance threshold of 0.01.

SCENIC (21) was a tool that utilizes scRNA-seq data to reconstruct gene regulatory networks. We used the pySCENIC (version 0.12.1) package in Python (version 3.10) to assess transcription factor enrichment and regulator activity of monocyte and nine macrophage subtypes. The grn function was used to infer gene co-expression relationships between transcription factors and their potential target. Then, ctx function was used to refine the regulons and separate direct and indirect target. Finally, the regulon activity was calculated by aucell function. The R package ComplexHeatmap (25) was used to visualize the regulon activity.

### 2.7 Signature genes

Based on cluster-specific marker genes, we identified the signature genes from FindAllMarkers function of Seurat (15). For macrophages, genes with adjusted *p value* < 0.01 by Wilcoxon rank-sum test, in a minimum of 50% of cells within the cluster and in a maximum of 50% of cells within the others cluster (pct.1 > 0.5 and pct.2 < 0.5), and demonstrating a differential expression threshold of 1 log fold change (avg_log2FC > 1) were defined as macrophage subtypes specific signature genes. For T cells, genes with adjusted *p value* < 0.01 by Wilcoxon rank-sum test, and demonstrating a differential expression threshold of 0.5 log fold change (avg_log2FC > 0.5) were defined as T cell subtypes specific signature genes. Then, we manually checked the cell subtypes specific signature genes. We removed ALB and ATP5F1E to confirm the signature genes were specific to cell subtypes. Furthermore, we performed SelectGene function of R package (26) (version 1.0) to calculate total entropy difference. The genes with greater total entropy difference tend to be more specific and would be retained as cell subtypes specific signature genes.

### 2.8 Survival analysis and prognostic gene selection

We collected 424 samples from the TCGA-LIHC cohort, including 50 normal liver samples and 374 HCC samples. Clinical information and gene expression matrix were retrieved using the R package TCGAbiolinks. The clinical information was retrieved using the GDCquery_clinic function. Bulk RNA-seq data were obtained using the GDCquery function with project set to “TCGA-LIHC”, data_category set to “Transcriptome Profiling”, data_type set to “Gene Expression Quantification”, and workflow_type set to “STAR–Counts. The TCGA-LIHC data was used to evaluate the prognostic performance of signature gene sets derived from macrophage and T cell subtypes clusters. We performed survival analysis using the Cox proportional hazards model implemented in the R package survival (version 3.3-1). And the ggsurvplot function was employed to correct patient age and plot Kaplan–Meier survival curves. Least absolute shrinkage and selection operator (LASSO) Cox regression analysis was applied to construct the prognostic model of the signature genes of SPP1^+^ Mac and Tc. Univariate Cox proportional hazards regression analysis was performed on the signature genes of SPP1^+^ Mac and Tc to screen genes significantly associated with OS in HCC (27).

### 2.9 Gene set enrichment score calculation

We employed ImmuCellAI (28) to calculate immune cell enrichment score (IS) of each sample to correct the bias of immune cell abundance. Hepatocytes can impair the function of T cell (29), so we calculated the enrichment score of Tc (CA) to assign the deviation. Next, we used the expression of signature gene set as input of ssGSEA to obtain the enrichment score (ES) of SPP1^+^ Mac and Tc. Finally, the gene set enrichment score are as follows:

Gene set enrichment score of SPP1^+^ Mac (Mscore):


$$\text{Mscore}=\frac{\text{ES}}{\text{IS}}$$

Gene set enrichment score of Tc (Tscore):


$$\text{Tscore}=\frac{\text{ES}*\text{CA}}{\text{IS}}$$

### 2.10 Mice

Experiments were conducted on 7–8 weeks old C57BL/6J male mice. Mice were purchased from the Hunan SJA Laboratory Animal Co., Ltd (Changsha, Hunan, China) and were bred in a specific pathogen‐free barrier facility at the Animal Center of Wuhan National Laboratory for Optoelectronics. 5 healthy mice as control. For Diet-induced MASH mice models, 5 mice were fed with MCD diet for up to 3 weeks. Liver fibrosis models were induced in 5 mice through an intraperitoneal injection of 25% CCL_4_ in corn oil (Sinopharm Chemical Reagent Co., Ltd., China) twice a week for 6 weeks. 5 HCC mice models generated by hydrodynamic tail vein injection of plasmids carrying the *Akt* and *N-ras* genes were established over six weeks and purchased from Shouzheng Pharma (Wuhan) Biotechnology Co., Ltd. Finally, all mice were anesthetized and transcardially perfused with RNase-free PBS. Liver samples of mice were immediately collected and subjected to further experiments.

### 2.11 Histology and FISH

H&E staining and Masson’s trichrome staining were used to evaluate and examine the histopathologic changes in liver structure. The sections were imaged with PanoBrain (Tinyphoton, Hubei, China). For each specimen, 5 fields per tissue section were randomly chosen and quantified by ImageJ software (National Institutes of Health, USA).

For FISH, the livers were maintained and fixed with 4% paraformaldehyde (PFA) for 6 hours, then incubated with 30% sucrose with 4% PFA. The liver lobes were immersed in optimal cutting temperature (OCT) tissue blocks, and stored at -80 °C. Cryosections 30 μm thick were used for *in situ* amplification and hybridization. Padlock probes and primers were designed as target sequence of genes (*C1qa, Cd3, Clec4f, Cxcl3, Cxcl10, Klf2, Trem2, Spp1, Akt and Nras*). In brief, the sections were incubated with buffer (2×SSC, 35% formamide, 1% TritonX-100 in RNase-free PBS) contain probes overnight at 37 °C. Then, 5.7 U/μl T4 DNA ligase (TaKaRa, Japan) was added to ligate padlock probes at room temperature for 4 hours. After ligation, the sections were incubated with rolling circle amplification mixture (250 μM dNTP, 4 μM Dithiothreitol, 0.4 U/μl Recombinant RNase Inhibitor in ddH_2_O) by Phi29 DNA polymerase (New England Biolabs, America) at 30°C for 2 hours. The sections were then incubated with monomer buffer (4% acrylamide, 0.2% bis-acrylamide, 0.1% ammonium persulfate, 0.2% tetramethylethylenediamine) at room temperature for 2 hours. Next, the sections were digested with Proteinase K (Biofrox, Germany) overnight in 37 °C. Finally, the sections were incubated with fluorescent oligo (Alexa Fluor 488, TAMRA, Alexa Fluor 647) complementary to DNA amplicon at 37 °C for 30 minutes. The sections were imaged on Olympus FLUOVIEW FV3000 confocal laser scanning microscopes for gene expression validations or cell subtypes analysis. The detailed probe information was listed on **Supplementary Table 4**.

### 2.12 Distance quantification of FISH data

The quantification of spatial distance was computed using cv2 python module. We used DAPI to stain the cell nucleus as the center point of the cell. We defined macrophages based on the fluorescent points of cell marker within the diameter of 20 μm, T cells were 10 μm. The FISH images were converted to the HSV color space, and which binary masks of valid regions were generated by applying specific thresholds. Next, the connected component analysis was performed on the masks to filter out small connected components and eliminate noise. The effective contours of fluorescent cells were extracted, and the distribution of their center points was calculated and visualized. For SPP1^+^ Mac and Tc, the shortest distance to a tumor cell was extracted from the pairwise cell-cell distance matrix of all cell distances, which defined as the distance to the closest tumor cell.

## 3 Results

### 3.1 A comprehensive cellular landscape of CLDs explored by integrated scRNA-seq data

**Figure 1:**
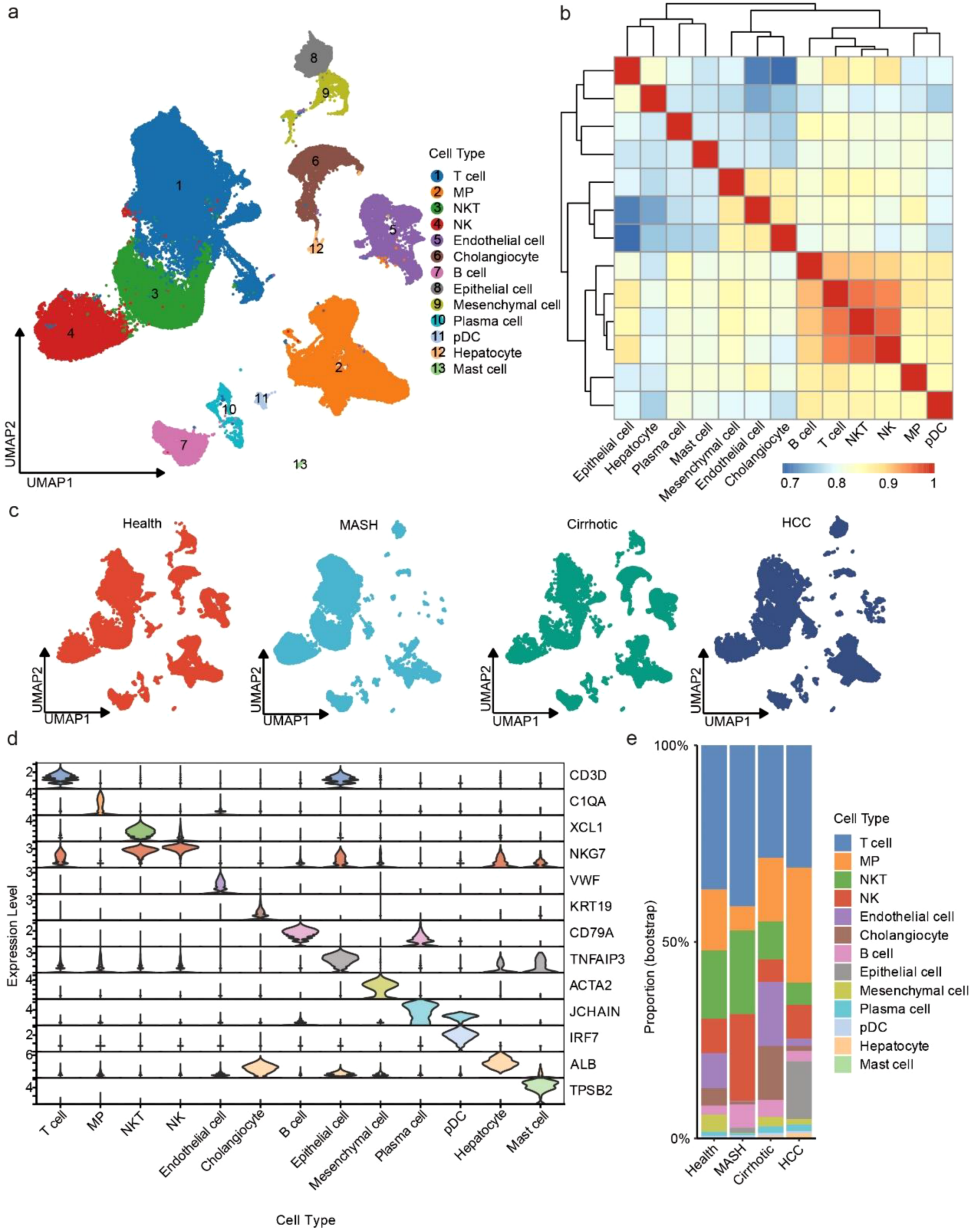
Single-cell RNA-seq landscape of major cell types at different processes of CLDs. **(a)** UMAP plot showing cell clusters in the progression of the chronic liver disease. MP, Mononuclear phagocyte; NK, natural killer T cell; NK, natural killer cell; pDC, plasmacytoid dendritic cell. **(b)** Spearman correlation of transcriptomic profiles of different cell populations. **(c)** UMAP plot colored by different progression of chronic liver diseases. **(d)** Violin plots showing the expressed marker genes of main cell types. **(e)** Histogram showing the proportion of main cell types in different progression of chronic liver diseases.

To uncovered the single-cell landscape during different processes of CLDs, we applied scRNA-seq data to characterize the dynamics from health liver, MASH, liver cirrhosis and HCC. In total, 99, 593 high-quality cells from 29 samples of 19 patients were analyzed after quality control in our study (**Supplementary Table 1**). After performing quality control, sctransform normalization, dimensionality reduction, batch effect removal, and clustering, the 13 distinct main cell types were annotated by CellTypist (17) and cell marker genes from CellMarker (30) (**Figure 1a**). We computed the spearman correlation of the main cell, and we observed a distinct correlation between adaptive immune cells (NKT and T cell) and innate immune cells (MP) and wanted to explore this further (**Figure 1b**). Each dataset with no obvious batch effects after batch correction (**Supplementary Figure 1a**). All cells with the number of genes per cell between 200 and 4000, and the proportion of mitochondrial gene counts less than 10% were selected (**Supplementary Figure 1b**). Of note, MP, T cell and NKT proportion were obviously dominant and distinct across different processes of CLDs, suggesting they might play a role in the immune microenvironment (**Figure 1c**). T cell expressed the T-cell receptor (TCR) signaling mediators CD3D (31), Mononuclear phagocyte (MP) was identified by the expression of C1QA, Natural killer T cell (NKT) was marked by XCL1 (32), Natural killer cell (NK) was identified by natural killer cell granule protein 7 (NKG7) (33), Endothelial cell was marked by known liver endothelial cell marker VWF (34), Cholangiocyte marker gene was KRT19 (35), B cell was identified by the canonical marker gene CD79A, Epithelial cell was defined by TNFAIP3 (36), Mesenchymal cell was marked by ACTA2 (37), Plasma cell was identified by the expression of JCHAIN (17), Plasmacytoid dendritic cell (pDC) was marked by IRF7 (38), Hepatocyte was defined by their classical marker ALB, Mast cell was positive for expression TPSB2 (39) (**Figure 1d**, **Supplementary Figure 1c**). To characterize the heterogeneous cellular compositions among the four processes of CLDs, we calculated the proportion of cell types by a bias-corrected and accelerated (BCa) bootstrap algorithm (40). Interestingly, we observed MPs and T cells (including T cells and NKT cells) proportion were distinct across different processes, which suggesting the progression heterogeneity of CLDs (**Figure 1e**). This indicated the complexity immune-specific microenvironment of CLDs (41). Hence, we proceeded to further annotated MPs and T cell subtypes with manual marker-based annotation method for in-depth analysis.

### 3.2 Intrahepatic macrophages and T cells subtypes exhibited distinct features during the progression of CLDs

**Figure 2:**
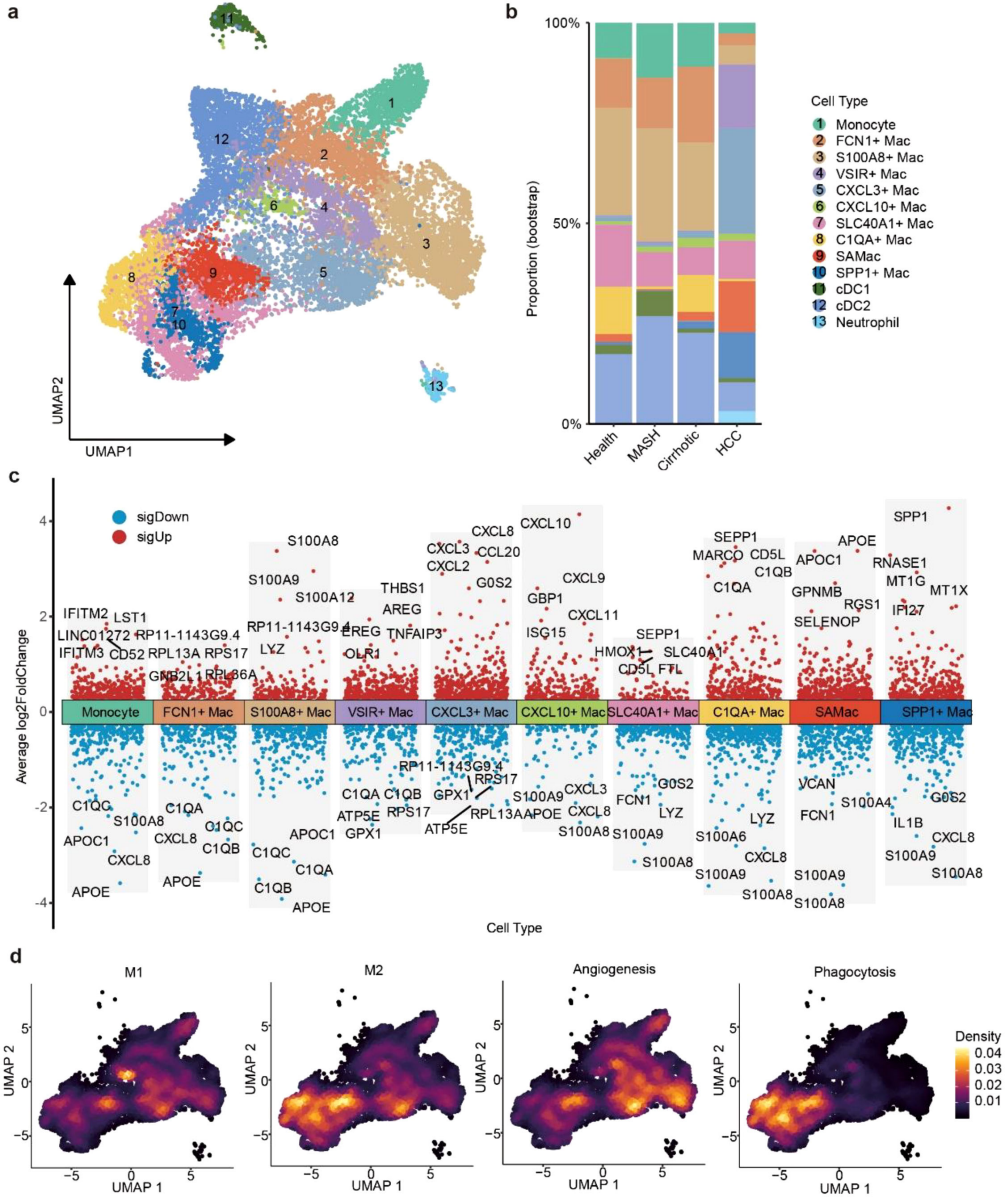
Characterization of macrophage subtypes and states during CLDs. **(a)** UMAP plot showing the MPs clusters, colors represent different cell populations, dots represent individual cells. cDC1, conventional dendritic cell 1; cDC2, conventional dendritic cell 2. **(b)** Histogram showing the proportion of MPs in different progression of CLDs. **(c)** Differential gene expression analysis showing up- (red) and down- (blue) regulated genes of monocytes and 9 macrophage subtypes. Adjusted p value < 0.05. **(d)** Density plot showing M1, M2, angiogenesis and phagocytosis properties of monocytes and 9 macrophage subtypes.

To pinpoint the transcriptional diversity in four processes of CLDs, we performed unsupervised clustering analyses of MPs. In addition to conventional dendritic cells (cDC1 and cDC2) and neutrophils, we annotated 10 other myeloid cell subtypes, including monocytes and 9 macrophage subtypes (**Figure 2a**). We found C1QA^+^ Mac highly enriched in healthy liver. SAMac and SPP1^+^ Mac was predominant in liver cirrhosis and HCC (**Figure 2b**). CXCL3^+^ Mac and CXCL10^+^ Mac highly expressed the genes of CXCL subfamily. CXCL3^+^ Mac exhibited the highest expression of CXCL8, CXCL3, CXCL2 and CCL20, which implied CXCL3^+^ Mac might be associated with liver disease progression and survival time (42, 43). CXCL10^+^ Mac was characterized by the inflammatory response chemokines of CXCL10 and CXCL9. It has been reported CXCL10 and CXCL9 were often localized with CXCL13-expressing T cells, which suggested they could participate in inflammation and antitumor reactivity (44). C1QA^+^ Mac highly expressed C1QA and C1QB, indicating that C1QA^+^ Mac might be involved in inhibiting tumor progression (45). SAMac specifically expressed TREM2, which was known to regulate scar-producing myofibroblasts (5). SPP1^+^ Mac was strikingly enriched in HCC and highest expressed immunosuppressive gene of *SPP1* (46) (**Figure 2c**).

We next evaluated the macrophage polarization states of M1, M2, angiogenesis and phagocytosis properties to infer their functional states (**Supplementary Figure 3a**; **Supplementary Table 2**). CXCL10^+^ Mac showed the highest M1 score, whereas CXCL3^+^ Mac showed the higher M2 and angiogenesis score, suggesting CXCL10^+^ Mac take part in chronic inflammation, CXCL3^+^ Mac was an immunosuppressive phenotype (22). C1QA^+^ Mac display the highest M2 and phagocytosis score, revealing that C1QA^+^ Mac might promote tolerance and diminished pro-inflammation (47). Notably, C1QA^+^ Mac also expressed the highest level of CD5L and MARCO, reported to be associated with anti-inflammation (48, 49), which implicating that C1QA^+^ Mac could be a beneficial cell population of CLDs (**Figures 2c, d**). SPP1^+^ Mac showed the lowest M1 score, supporting SPP1^+^ Mac contributors to pro-tumor (50). Our observations elucidated the inflammatory response of CXCL3^+^ Mac, CXCL10^+^ Mac and C1QA^+^ Mac, and revealing SPP1^+^ Mac predominant present in HCC, implicating their important roles during CLDs processes.

We identified 14 T cell subtypes by unsupervised clustering analysis of T cells and NK cells (**Supplementary Figure 2a**), including naive T cell (Tn), NKT, exhausted T cell (Tex), effector T cell (Teff), proliferating T cell (Tproli), T helper cell (Th), T regulatory cell (Treg), stress response T cell (Tstr), effector memory T cell (Tem), cytotoxic T cell (Tc), Cycling NK & T, gamma delta T cell (γδT), mucosal-associated invariant T cell (MAIT) and tissue-resident memory T cell (Trm). But we didn’t observe changing patterns in T cell compositions during CLDs processes (**Supplementary Figure 2b**). Tn expressed high levels of naive markers. Tex demonstrated the highest expression of CXCL13. Tstr characterized by high expression of heat shock genes. Tc markedly expressed cytolytic activity-related genes. Tem showed high expression of immunoglobulin-related genes (**Supplementary Figure 2c**). To understand the state of T cells, we calculated the scores of naive, activation, cytotoxicity, and exhaustion onto the UMAP, which was following their expected functions (**Supplementary Figure 2d**; **Supplementary Table 3**).

### 3.3 The trajectories and regulators of macrophages and T cells subtypes in different CLDs processes

**Figure 3:**
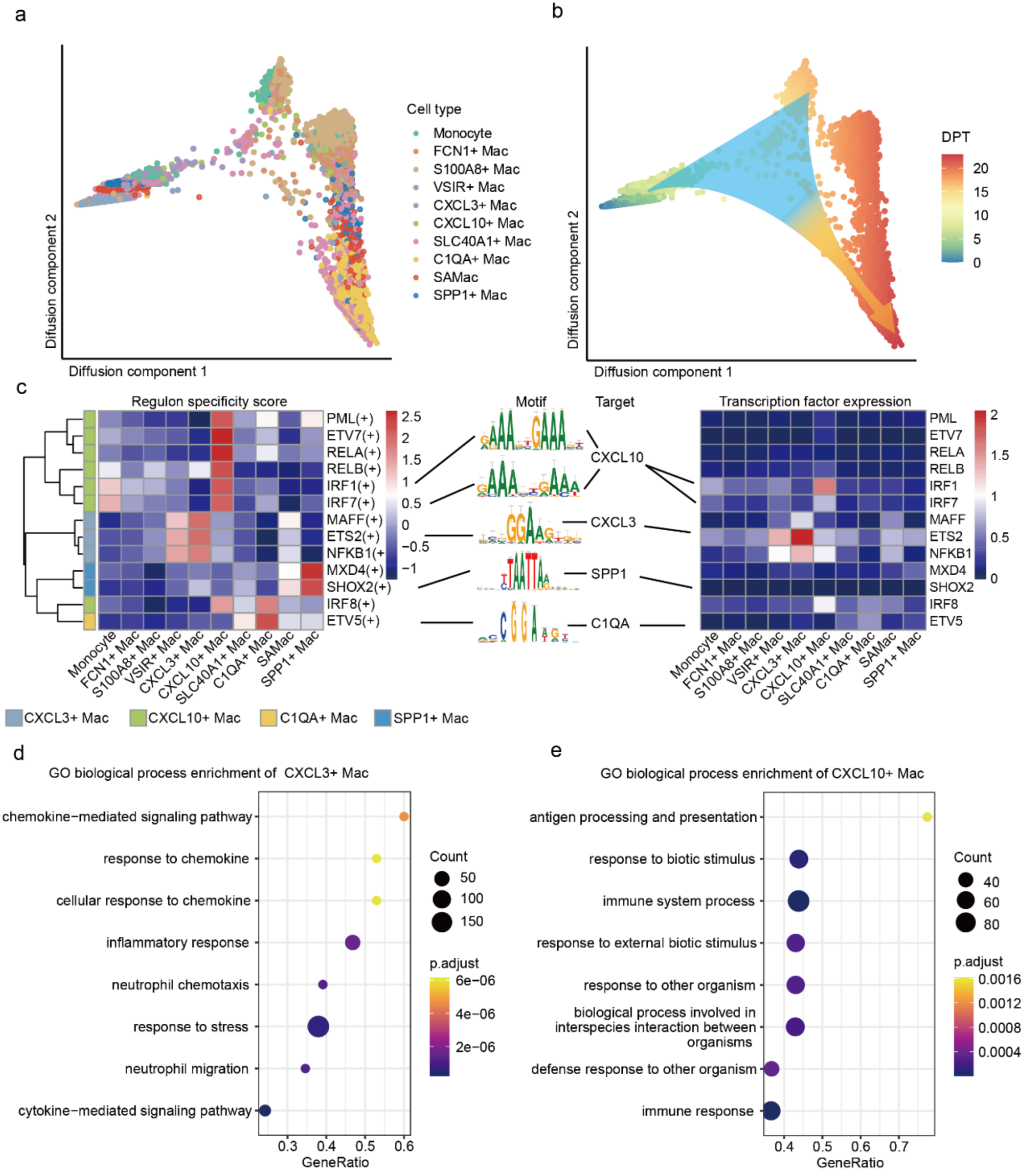
Developmental trajectory and of transcription factor activity analysis of macrophage subtypes during the progression of CLDs. **(a)** Confirmation of trajectories using a diffusion-map approach. **(b)** Pseudo-time trajectory projected of macrophage subtypes. Pseudo-time values were color coded. DPT, diffusion pseudo time. **(c)** Heatmap showing the regulon activities of transcription factors (left) and expression profiles (right) in different macrophage subtypes; the color key from blue to red indicated regulon specificity score and relative expression levels from low to high. **(d, e)**. Dot plots showing the enrichment of the biological process-related GO terms in CXCL3^+^ Mac **(d)** and CXCL10^+^ Mac **(e)**.

The CLDs processes were dynamic (51). To reveal novel cellular and molecular mechanisms driving MASH to HCC, we further explored the differentiation trajectories and transcriptional regulation of macrophages and T cells subtypes. We applied Destiny (19) to infer the differentiation trajectory of macrophages and T cells subtypes. Macrophages subtypes displayed a trajectory that started with monocytes. FCN1^+^ Mac, S100A8^+^ Mac, and VSIR^+^ Mac were primary site in initial state same as monocyte. CXCL3^+^ Mac and CXCL10^+^ Mac mainly in intermediate state, which might drive the unique transcriptomic identities of MASH microenvironment (52). C1QA^+^ Mac, SAMac and SPP1^+^ Mac were dominating located in terminal state (**Figures 3a, b**), SAMac has been proved accumulation within the fibrotic niche (5). These findings supported that the HCC was correlated with C1QA^+^ Mac and SPP1^+^ Mac. T cells displayed two paths started with Tn. The path 1 ending in a terminally differentiation of Tex, Tc, γδT and Trm. The path 2 contained Tn, Treg and MAIT (**Supplementary Figures 4b, c**). These suggested the path 1 of T cells might be associated with HCC (23).

To investigate the transcriptional regulation dynamics of different macrophage subtypes during CLDs progression, we applied SCENIC (21) to calculate the activity of cell-type-specific regulators across CLDs processes. Interestingly, we found various transcription factors were highly expressed within the corresponding macrophage subtypes. The expression of *ETS2* and *IRF1* were consisted with transcription factors activities, which suggested that *ETS2/IRF1* was specific targeting to CXCL3^+^ Mac/CXCL10^+^ Mac (**Figure 3c**; **Supplementary Figure 4a**). In addition, we explored gene ontology (GO) analyses to reveal CXCL3^+^ Mac was enriched in chemokine−mediated signaling pathway and inflammatory response (**Figure 3d**), CXCL10^+^ Mac was significantly enriched in response to biotic stimulus and immune response (**Figure 3e**). These implied that CXCL3^+^ Mac and CXCL10^+^ Mac might play important roles in MASH.

### 3.4 Validation of the macrophage subtypes compositions using mouse models

**Figure 4:**
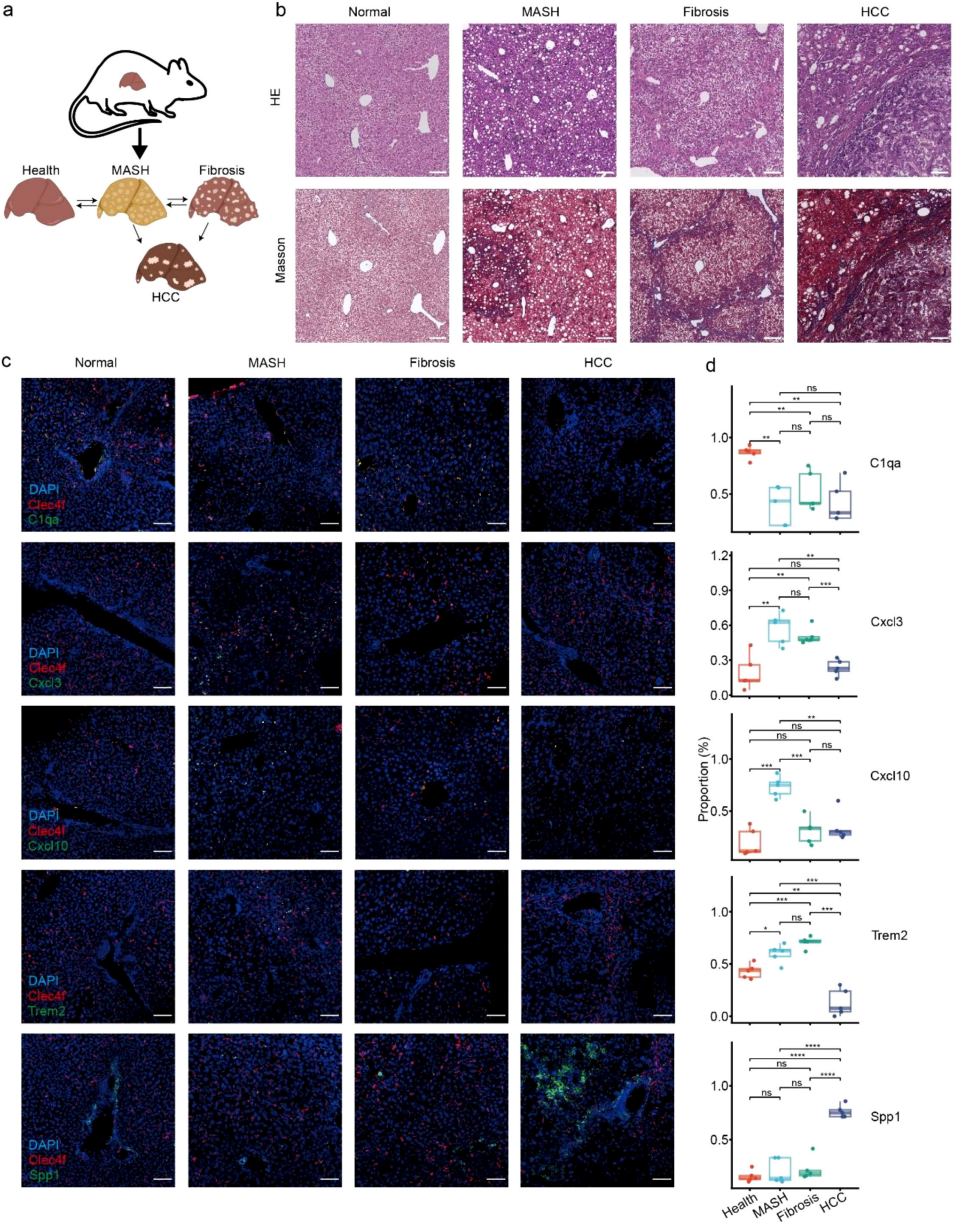
Validation of the macrophage subtypes abundance with healthy liver, MASH, liver fibrosis, and HCC mice models. **(a)** Schematic representation of the mouse models. **(b)** Histological observation of Healthy liver, MASH, liver fibrosis and HCC by H&E and Masson’s trichrome staining. Images were representative of three biologically independent mice. Scale bar: 200 µm. **(c)** FISH staining to validate the macrophage subtypes compositions. Scale bars: 200 µm. **(d)** The macrophage subtypes compositions across chronic liver disease progressions calculated from **Figure 4c** (n = 5). Two-sided Student’s t test. Statistical significance was indicated as follows: p < 0.05 (*), p < 0.01 (**), p < 0.001 (***), and p < 0.0001 (****), ns, not significant.

To validate the compositions of macrophage subtypes across CLDs progresses from scRNA-seq analysis, we constructed the mouse models of MASH, liver fibrosis and HCC (**Figure 4a**). Firstly, we evaluated the inflammation and fibrosis at the histological level by H&E and Masson’s trichrome staining to confirm the results in the scRNA-seq analysis (**Figure 4b**). Next, we utilized FISH to visualize those macrophage subtypes and directly calculated their cellular abundances. We used *Clec4f* as canonical liver macrophage marker. The well-conserved genes of *C1qa, Cxcl3, Cxcl10, Trem2* and *Spp1* were used to mark the signature genes (**Figure 4c**; **Supplementary Table 4**). If the signal of macrophage subtype signature genes were within 20 microns in diameter of Clec4f signal at 400 µm × 400 µm region, we recognized they were the corresponding macrophage subtype. As a result, C1QA^+^ Mac was enriched in healthy liver, CXCL3^+^ Mac and CXCL10^+^ Mac were commonly present in MASH, SAMac was enriched in liver fibrosis, and SPP1^+^ Mac was predominantly present in HCC (**Figures 4d, e**). Collectively, we affirmed the 5 macrophage subtypes presented at different processes of CLDs progresses.

### 3.5 Macrophages-T cells interactions revealed hetero-cellular crosstalk in CLDs progresses

**Figure 5:**
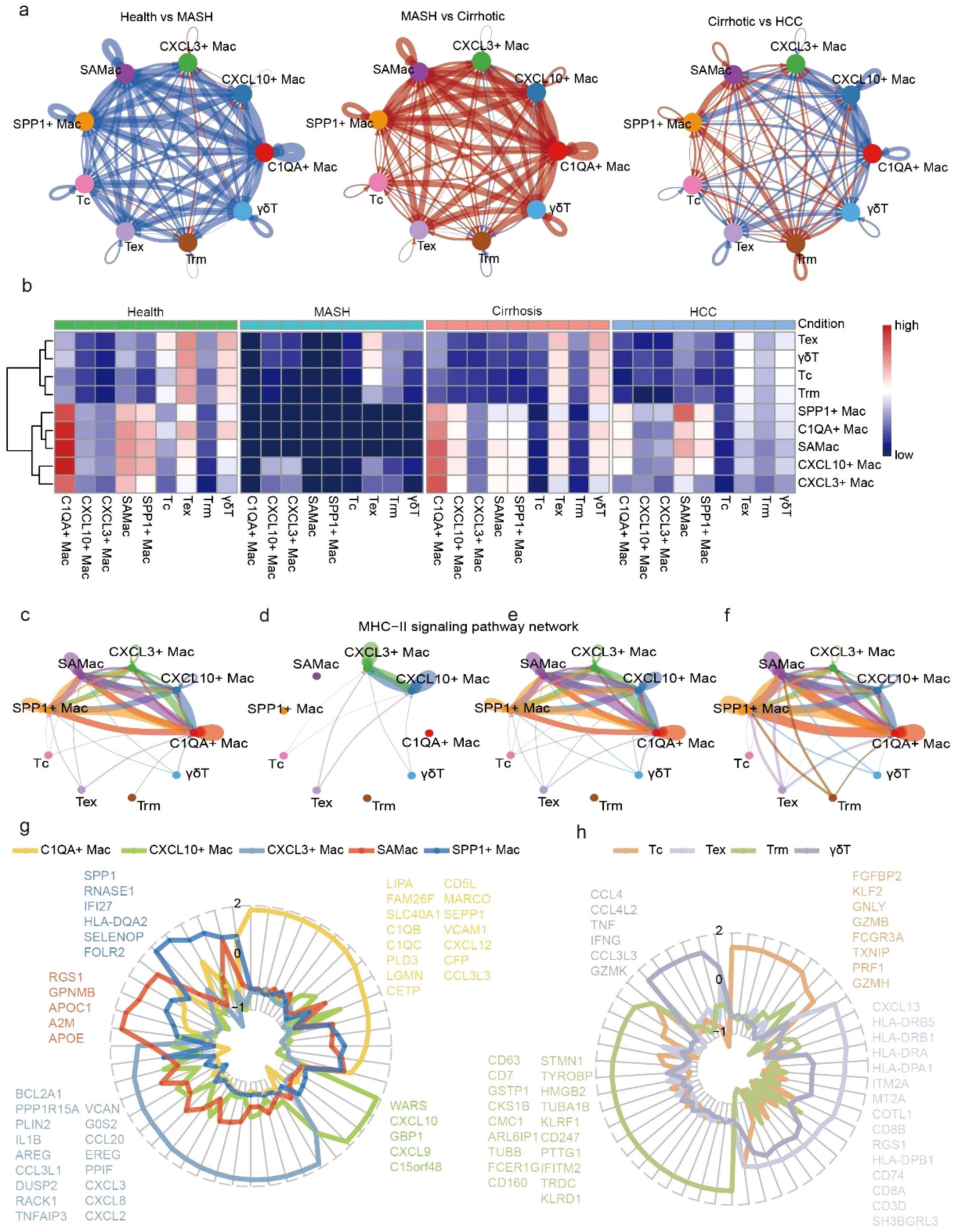
The intercellular communication and signature genes of disease associated T cell and macrophage in different disease processes. **(a)** Circos plot showed differential interaction strength relative in healthy liver, MASH, liver cirrhosis and HCC, red indicated increased interaction strength and blue indicated decreased strength. **(b)** Heatmap showed interaction strength among 9 cell subtypes in chronic liver disease progressions, red represented increased interactive strength, blue represented decreased interactive strength. c-f. The MHC-II signaling pathway network in healthy liver **(c)**, MASH **(d)**, liver cirrhosis **(e)** and HCC **(f)**. **(g)** Radar charts showed the entropy difference of gene in C1QA^+^ Mac, CXCL3^+^ Mac, CXCL10^+^ Mac, SAMac and SPP1^+^ Mac. **(h)** Radar charts showed the entropy difference of gene in Tc, Tex, Trm and γδT.

We next aimed to understand the complex cellular interactions among diseases-associated macrophages and T cells. CellChat (20) was used to compare macrophages-T cells interactions in the previous versus subsequent processes of CLDs. The comparisons of macrophages-T cells interactions were distinct differences during the progression of CLDs. We observed CXCL3^+^ Mac and CXCL10^+^ Mac showed stronger crosstalks with Tex, Trm and γδT between health and MASH processes. The interaction strength of SAMac and SPP1^+^ Mac were increased in both MASH versus cirrhosis and cirrhosis versus HCC. C1QA^+^ Mac showed strong interaction strength in healthy liver and from MASH to cirrhosis (**Figures 5a, b**). MHC class II (MHC-II) were constitutively expressed on the surface of macrophages. MHC-II complexes depended on the distinct macrophage subtypes (53). We inferred cell-cell communication at the signaling pathway level. The interactions between CXCL3^+^ Mac and CXCL10^+^ Mac were mediated mainly by MHC-II signaling pathway. The interactions of MHC-II signaling pathway increased significantly from C1QA^+^ Mac, SAMac and SPP1^+^ Mac in healthy liver and HCC (**Figures 5c-f**). To further elucidated the molecular characteristics of diseases-associated macrophages and  T cells, we utilized entropy test to detect the signature gene sets from the differentially expressed genes of each cell subtype (**Figures 5g, h**). Notably, we found *SPP1, IFI27, FOLR2* and *SELENOP* were the signature gene sets of SPP1^+^ Mac. These genes were reported to participate in immunosuppression and influence liver metabolic activites (12, 54, 55). The signature gene sets of Tc contained cytotoxicity-related molecules (*FGFBP2, GNLY, GZMB, PRF1* and *GZMH*) and the transcription factor of suppress exhaustion (*KLF2*) (56, 57). These results indicated that SPP1^+^ Mac and Tc could be potential targets for HCC immunotherapy. We want to further identify the potential therapeutic targets from the signature gene sets. In brief, our results suggested a differentiated macrophages-T cells interactions and molecular phenotypes during the progression of CLDs.

### 3.6 Integration of bulk RNA-seq data to revealed predictive prognostic biomarkers in HCC

**Figure 6:**
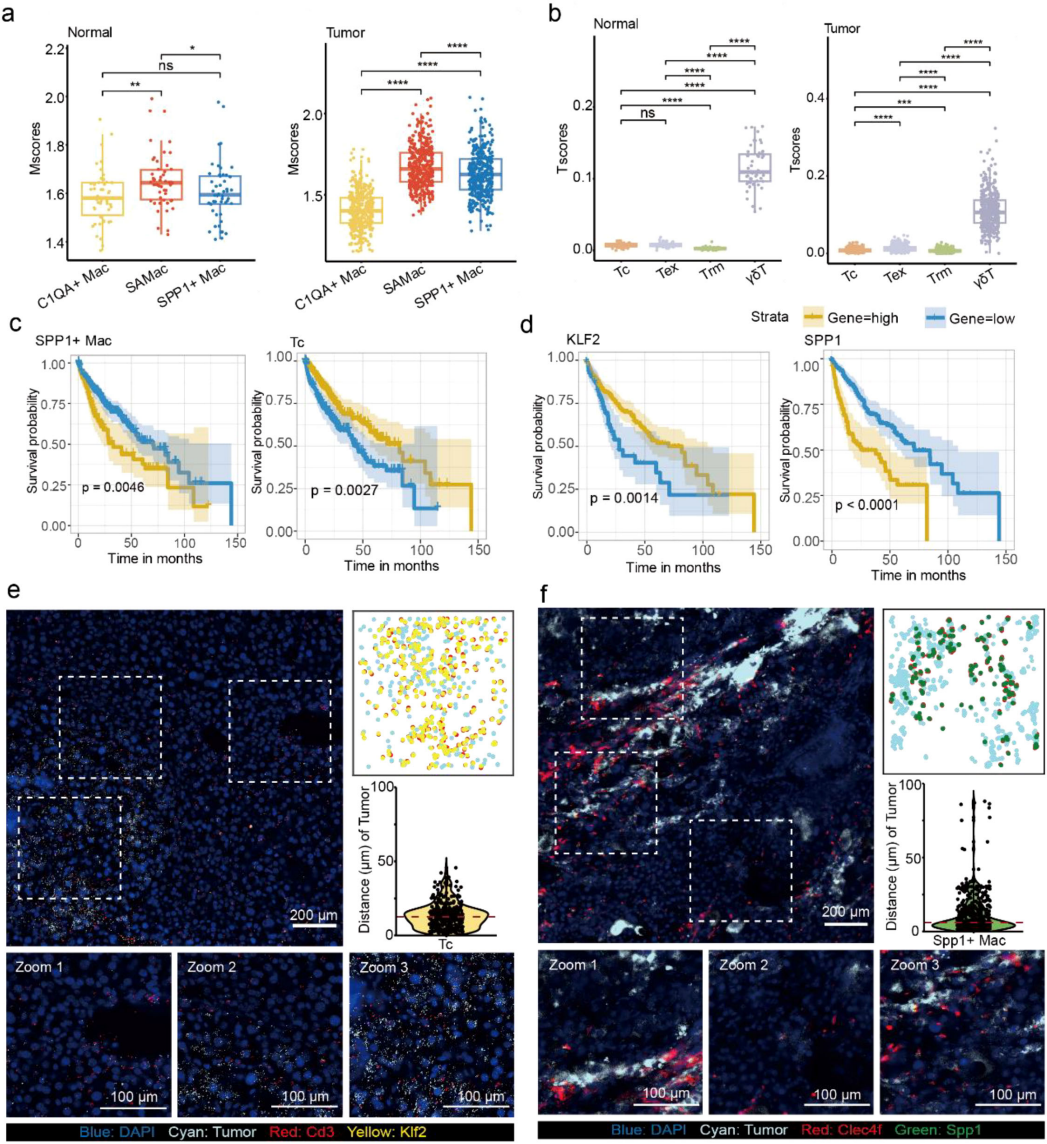
KLF2 contributed to the good and SPP1 induced bad clinical outcome in HCC. **(a)** Boxplot showing the Mscores of macrophage subtypes in healthy liver (n = 50) and HCC (n = 374). Wilcoxon test. **(b)** Boxplot showing the Tscores of T cell subtypes in healthy liver (n = 50) and HCC (n = 374). Wilcoxon test. **(c)** Kaplan-Meier plots showing clinical outcome in HCC with Mscore of SPP1^+^ Mac and Tscore of Tc. **(d)** Kaplan-Meier plots showing clinical outcome in HCC with the expression of *SPP1* and *KLF2* in HCC. **(e, f)**. Left: Representative FISH micrographs (n ≥ 3) showed the distribution of Tc **(e)** and SPP1^+^ Mac **(f)** in HCC. Close-up images on the (left bottom) correspond to boxed regions on the (top left). Top right: Scatterplots showed the distribution of SPP1^+^ Mac and Tc corresponding to FISH micrographs on the Zoom 3. Bottom right: Violin plots showed the distance quantification of SPP1^+^ Mac and Tc to the closest tumor cell corresponding cells identified on FISH micrographs on Zoom 3. *Spp1* (green), *Klf2* (yellow), *Cd3* and *Clec4f* (red), HCC (cyan) and DAPI (blue). Statistical significance was indicated as follows: p < 0.05 (*), p < 0.01 (**), p < 0.001 (***), and p < 0.0001 (****), ns, not significant.

ScRNA-seq provided an insight on the cellular heterogeneity in the TME. However, the scRNA-seq studies lacked survival data. We employed TCGA-LIHC data to verify the expression patterns of signature gene sets in HCC and reveal the key genes contributing to HCC. We developed a method of gene sets enrichment score to validate the signature gene sets from scRNA-seq (**Figures 5g, h**) based on TCGA-LIHC dataset. We calculated gene sets enrichment score of macrophage subtypes and defined as Mscore. The Tscore was defined for gene sets enrichment score of T cell subtypes. The Mscore and Tscore were extensions of the ImmuCellAI (28), designed to provide a simple and intuitive summary of predefined immune gene sets derived from scRNA-seq data, enabling the comparison of estimated cell populations in HCC. The details of the method calculating gene sets enrichment score were described in Methods. We found Mscore of SPP1^+^ Mac and Tscore of Tc were significant difference in the HCC group than the normal group (**Figures 6a, b**), which further validating SPP1^+^ Mac and Tc were associated with HCC. Next, basing on the clinical data from the TCGA-LIHC project, we confirmed that the higher Tscore of Tc was associated with a survival advantage in HCC, while an opposite effect was observed in SPP1^+^ Mac (**Figure 6c**), consistent with our hypothesis. Furthermore, we employed LASSO regression model and Cox regression analysis to reveal *KLF2* was contributed to favorable prognosis and *SPP1* were associated with poor outcomes in HCC (**Figure 6d**; **Supplementary Figures 6a, b**). As reported, *KLF2* was favored effector differentiation and suppressed exhaustion (58). *SPP1* overexpression was identified in tumor-associated macrophages across several cancer types and associated with poor prognosis (59).

In addition, we revealed the spatial distribution and cellular interaction of SPP1^+^ Mac and Tc. We quantified distance from SPP1^+^ Mac and Tc to its closest HCC cell by FISH micrographs. **Figures 6e, f** showed the FISH micrographs of the markers (top left, **Supplementary Table 4**), the corresponding dot plots of Zoom 3 representing the spatial distribution of SPP1^+^ Mac and Tc (bottom left), and the corresponding distance quantification from SPP1^+^ Mac and Tc to the closest HCC cell in Zoom 3 (bottom right). We found that Tc localized further away from the HCC cell (**Figure 6e** and **Supplementary Figure 6c**, with a median distance of 12.6 μm) compared to SPP1^+^ Mac that infiltrated and tightly surrounded the HCC cell (**Figure 6f** and **Supplementary Figure 6c**, with a median distance of 6.1 μm). The FISH micrographs also demonstrated that the number of SPP1^+^ Mac and Tc were significantly higher in HCC, and the double-positive cell pairs of *Spp1* with *Clec4f* and *Klf2* with *Cd3* were close proximity to that in healthy liver (**Supplementary Figures 6d-g**, with a median distance of 0 μm). It suggested the potential crosstalk between SPP1^+^ Mac and Tc. Taken together, these findings suggested SPP1^+^ Mac contributed to the immunosuppressive microenvironment in HCC, Tc played anti-tumorigenic roles of HCC. We also determined that *KLF2* and *SPP1* involved in the progression of HCC and could be further clinical investigation.

## 4 Discussion

MASH now became the fastest growing cause of liver cancer; however, the increased risk of HCC in patients with MASH was often misdiagnosed (51). Our study deciphered the molecular signatures and functional properties of the immune cells at different stages of CLD processes, rather than a linear evolutionary trajectory of NASH-induced chronic liver disease. By integrating scRNA-seq, bulk RNA-seq and FISH, we elucidated diverse cellular populations, phenotypic states and transcriptional profiles, which providing molecular characteristics for clinical diagnosis and immunotherapy of CLDs. Because mRNA and protein expression levels could differ substantially and were not necessarily linearly correlated, we employed FISH to validate the molecular markers at the transcriptional level. Future validation at the protein level using immunofluorescence or flow cytometry could strengthen these findings.

It has been recognized that both innate and adaptive immune mechanisms played important roles in promoting hepatic inflammation of MASH-HCC (8). Focused on macrophages and T cells, we identified five macrophage subtypes (CXCL3^+^ Mac, CXCL10^+^ Mac, C1QA^+^ Mac, SPP1^+^ Mac and SAMac) and four T cell subtypes (Tex, Tc, γδT and Trm) associated with CLDs processes. We overcame the limitations of single-disease data and identified stage-specific cell types by integrating data from multiple stages of CLDs. The stage-specific macrophage subtypes exhibited diverse patterns. The lipid-associated macrophage has been reported to arise prominently under obesity conditions in adipose tissue, highlighting the specific expression of *Trem2* (60). However, it resembled the SAMac in liver cirrhosis (5). We found CXCL3^+^ Mac and CXCL10^+^ Mac were enriched in MASH and resided in intermediate stages of CLDs progression, with *ETS2* and *IRF1* potentially involved in regulating their transcriptional programs, which might potentially contribute to the transition from MASH to HCC. *ETS2* has been reported as a central regulator of macrophage inflammation (61). *IRF1* could regulate the transcription of inflammation and cell death related genes, which induced further elicited inflammatory injury (62). C1QA^+^ Mac, SPP1^+^ Mac and SAMac displayed terminal-stage characteristics. Together, these findings suggested the distinct cellular heterogeneity gave rise to phenotypic and functional diversity, which in turn shaped microenvironmental variation in CLDs progression. A limitation of these results was supported at the transcriptomic level based on FISH; further validation at the protein level using immunofluorescence or flow cytometry could strengthen these findings.

Previous studies have reported SPP1^+^ Mac could induce exhaustion and dysfunction of tumor-reactive CD8 ^+^ T cell in extrahepatic tumors (63), while their effects on T cell subtype responses in HCC remained unclear. Notably, we uncovered SPP1^+^ Mac was predominant in HCC and correlated with poor prognosis, potentially driving immune evasion by establishing anergic crosstalk with Tc. In addition, we constructed gene sets enrichment score to detect *KLF2* in Tc and *SPP1* in SPP1^+^ Mac as HCC prognosis-related genes. *KLF2* has been reported to improve the clinical benefit in HCC (64), whereas *SPP1* was associated with worse overall survival in macrophage (46). We first found the closely interaction between SPP1^+^ Mac and Tc in HCC, suggesting that SPP1^+^ Mac could directly suppress the activation of Tc in HCC microenvironment, with *KLF2* and *SPP1* as key molecular biomarkers.

In conclusion, our work constructed the transcriptional atlas of CLD progression at single cell level, which might contribute to understanding molecular characteristics for clinical diagnosis and immunotherapy of CLDs. We revealed stage-specific macrophage subtypes and identified *ETS2/IRF1* regulated macrophage programs driving MASH to HCC transition. Additionally, we combined the signature gene sets from scRNA-seq with the TCGA-LIHC to resolve *SPP1* and *KLF2* were the HCC associated genes, which might provide relevant therapeutic targets.

## Data Availability

The original contributions presented in the study are included in the article/**Supplementary Material**. Further inquiries can be directed to the corresponding authors.

## References

[1] Cheemerla S Balakrishnan M . Global epidemiology of chronic liver disease. Clin Liver Dis. (2021) 17:365–70. doi: 10.1002/cld.1061, PMID: 34136143 PMC8177826

[2] Manikat R Ahmed A Kim D . Current epidemiology of chronic liver disease. Gastroenterol Rep. (2023) 12:goae069. doi: 10.1093/gastro/goae069, PMID: 38915345 PMC11194530

[3] Marcellin P Kutala BK . Liver diseases: A major, neglected global public health problem requiring urgent actions and large-scale screening. Liver Int. (2018) 38:2–6. doi: 10.1111/liv.13682, PMID: 29427496

[4] Huang DQ Terrault NA Tacke F Gluud LL Arrese M Bugianesi E . Global epidemiology of cirrhosis — aetiology, trends and predictions. Nat Rev Gastroenterol Hepatol. (2023) 20:388–98. doi: 10.1038/s41575-023-00759-2, PMID: 36977794 PMC10043867

[5] Ramachandran P Dobie R Wilson-Kanamori JR Dora EF Henderson BEP Luu NT . Resolving the fibrotic niche of human liver cirrhosis at single-cell level. Nature. (2019) 575:512–8. doi: 10.1038/s41586-019-1631-3, PMID: 31597160 PMC6876711

[6] Pfister D Núñez NG Pinyol R Govaere O Pinter M Szydlowska M . NASH limits anti-tumour surveillance in immunotherapy-treated HCC. Nature. (2021) 592:450–6. doi: 10.1038/s41586-021-03362-0, PMID: 33762733 PMC8046670

[7] Pinyol R Torrecilla S Wang H Montironi C Piqué-Gili M Torres-Martin M . Molecular characterisation of hepatocellular carcinoma in patients with non-alcoholic steatohepatitis. J Hepatol. (2021) 75:865–78. doi: 10.1016/j.jhep.2021.04.049, PMID: 33992698 PMC12164395

[8] Llovet JM Willoughby CE Singal AG Greten TF Heikenwälder M El-Serag HB . Nonalcoholic steatohepatitis-related hepatocellular carcinoma: pathogenesis and treatment. Nat Rev Gastroenterol Hepatol. (2023) 20:487–503. doi: 10.1038/s41575-023-00754-7, PMID: 36932227 PMC12165718

[9] Remmerie A Martens L Thoné T Castoldi A Seurinck R Pavie B . Osteopontin expression identifies a subset of recruited macrophages distinct from kupffer cells in the fatty liver. Immunity. (2020) 53:641–657.e14. doi: 10.1016/j.immuni.2020.08.004, PMID: 32888418 PMC7501731

[10] Zhang Q He Y Luo N Patel SJ Han Y Gao R . Landscape and dynamics of single immune cells in hepatocellular carcinoma. Cell. (2019) 179:829–845.e20. doi: 10.1016/j.cell.2019.10.003, PMID: 31675496

[11] Tian Z Xu C Yang P Lin Z Wu W Zhang W . Molecular pathogenesis: Connections between viral hepatitis-induced and non-alcoholic steatohepatitis-induced hepatocellular carcinoma. Front Immunol. (2022) 13:984728. doi: 10.3389/fimmu.2022.984728, PMID: 36189208 PMC9520190

[12] Liu Y Xun Z Ma K Liang S Li X Zhou S . Identification of a tumour immune barrier in the HCC microenvironment that determines the efficacy of immunotherapy. J Hepatol. (2023) 78:770–82. doi: 10.1016/j.jhep.2023.01.011, PMID: 36708811

[13] Lun ATL Riesenfeld S Andrews T Dao TP Gomes T participants in the 1st Human Cell Atlas Jamboree . EmptyDrops: distinguishing cells from empty droplets in droplet-based single-cell RNA sequencing data. Genome Biol. (2019) 20:63. doi: 10.1186/s13059-019-1662-y, PMID: 30902100 PMC6431044

[14] McGinnis CS Murrow LM Gartner ZJ . DoubletFinder: doublet detection in single-cell RNA sequencing data using artificial nearest neighbors. Cell Syst. (2019) 8:329–337.e4. doi: 10.1016/j.cels.2019.03.003, PMID: 30954475 PMC6853612

[15] Hao Y Hao S Andersen-Nissen E Mauck WM Zheng S Butler A . Integrated analysis of multimodal single-cell data. Cell. (2021) 184:3573–3587.e29. doi: 10.1016/j.cell.2021.04.048, PMID: 34062119 PMC8238499

[16] Korsunsky I Millard N Fan J Slowikowski K Zhang F Wei K . Fast, sensitive and accurate integration of single-cell data with Harmony. Nat Methods. (2019) 16:1289–96. doi: 10.1038/s41592-019-0619-0, PMID: 31740819 PMC6884693

[17] Domínguez Conde C Xu C Jarvis LB Rainbow DB Wells SB Gomes T . Cross-tissue immune cell analysis reveals tissue-specific features in humans. Science. (2022) 376:eabl5197. doi: 10.1126/science.abl5197, PMID: 35549406 PMC7612735

[18] Hu C Li T Xu Y Zhang X Li F Bai J . CellMarker 2.0: an updated database of manually curated cell markers in human/mouse and web tools based on scRNA-seq data. Nucleic Acids Res. (2023) 51:D870–6. doi: 10.1093/nar/gkac947, PMID: 36300619 PMC9825416

[19] Angerer P Haghverdi L Büttner M Theis FJ Marr C Buettner F . destiny: diffusion maps for large-scale single-cell data in R. Bioinformatics. (2016) 32:1241–3. doi: 10.1093/bioinformatics/btv715, PMID: 26668002

[20] Jin S Guerrero-Juarez CF Zhang L Chang I Ramos R Kuan C-H . Inference and analysis of cell-cell communication using CellChat. Nat Commun. (2021) 12:1088. doi: 10.1038/s41467-021-21246-9, PMID: 33597522 PMC7889871

[21] Aibar S González-Blas CB Moerman T Huynh-Thu VA Imrichova H Hulselmans G . SCENIC: single-cell regulatory network inference and clustering. Nat Methods. (2017) 14:1083–6. doi: 10.1038/nmeth.4463, PMID: 28991892 PMC5937676

[22] Wang R Song S Qin J Yoshimura K Peng F Chu Y . Evolution of immune and stromal cell states and ecotypes during gastric adenocarcinoma progression. Cancer Cell. (2023) 41:1407–1426.e9. doi: 10.1016/j.ccell.2023.06.005, PMID: 37419119 PMC10528152

[23] Chu Y Dai E Li Y Han G Pei G Ingram DR . Pan-cancer T cell atlas links a cellular stress response state to immunotherapy resistance. Nat Med. (2023) 29:1550–62. doi: 10.1038/s41591-023-02371-y, PMID: 37248301 PMC11421770

[24] Xu S Hu E Cai Y Xie Z Luo X Zhan L . Using clusterProfiler to characterize multiomics data. Nat Protoc. (2024) 19:3292–320. doi: 10.1038/s41596-024-01020-z, PMID: 39019974

[25] Gu Z . Complex heatmap visualization. iMeta. (2022) 1:e43. doi: 10.1002/imt2.43, PMID: 38868715 PMC10989952

[26] Li C Liu B Kang B Liu Z Liu Y Chen C . SciBet as a portable and fast single cell type identifier. Nat Commun. (2020) 11:1818. doi: 10.1038/s41467-020-15523-2, PMID: 32286268 PMC7156687

[27] He L Chen J Xu F Li J Li J . Prognostic implication of a metabolism-associated gene signature in lung adenocarcinoma. Mol Ther Oncolyt. (2020) 19:265–77. doi: 10.1016/j.omto.2020.09.011, PMID: 33209981 PMC7658576

[28] Miao Y Zhang Q Lei Q Luo M Xie G Wang H . ImmuCellAI: A unique method for comprehensive T-cell subsets abundance prediction and its application in cancer immunotherapy. Adv Sci. (2020) 7:1902880. doi: 10.1002/advs.201902880, PMID: 32274301 PMC7141005

[29] Kawashima K Andreata F Beccaria CG Iannacone M . Priming and maintenance of adaptive immunity in the liver. Annu Rev Immunol. (2024) 42:375–99. doi: 10.1146/annurev-immunol-090122-041354, PMID: 38360545

[30] Zhang X Lan Y Xu J Quan F Zhao E Deng C . CellMarker: a manually curated resource of cell markers in human and mouse. Nucleic Acids Res. (2019) 47:D721–8. doi: 10.1093/nar/gky900, PMID: 30289549 PMC6323899

[31] Wang X Yang L Wang Y-C Xu Z-R Feng Y Zhang J . Comparative analysis of cell lineage differentiation during hepatogenesis in humans and mice at the single-cell transcriptome level. Cell Res. (2020) 30:1109–26. doi: 10.1038/s41422-020-0378-6, PMID: 32690901 PMC7784864

[32] Young MD Mitchell TJ Vieira Braga FA Tran MGB Stewart BJ Ferdinand JR . Single-cell transcriptomes from human kidneys reveal the cellular identity of renal tumors. Science. (2018) 361:594–9. doi: 10.1126/science.aat1699, PMID: 30093597 PMC6104812

[33] Ng SS De Labastida Rivera F Yan J Corvino D Das I Zhang P . The NK cell granule protein NKG7 regulates cytotoxic granule exocytosis and inflammation. Nat Immunol. (2020) 21:1205–18. doi: 10.1038/s41590-020-0758-6, PMID: 32839608 PMC7965849

[34] Wang X He Y Zhang Q Ren X Zhang Z . Direct comparative analyses of 10X genomics chromium and smart-seq2. Genom Proteomics Bioinf. (2021) 19:253–66. doi: 10.1016/j.gpb.2020.02.005, PMID: 33662621 PMC8602399

[35] Montal R Sia D Montironi C Leow WQ Esteban-Fabró R Pinyol R . Molecular classification and therapeutic targets in extrahepatic cholangiocarcinoma. J Hepatol. (2020) 73:315–27. doi: 10.1016/j.jhep.2020.03.008, PMID: 32173382 PMC8418904

[36] Anderson CJ Medina CB Barron BJ Karvelyte L Aaes TL Lambertz I . Microbes exploit death-induced nutrient release by gut epithelial cells. Nature. (2021) 596:262–7. doi: 10.1038/s41586-021-03785-9, PMID: 34349263

[37] Govaere O Cockell S Tiniakos D Queen R Younes R Vacca M . Transcriptomic profiling across the nonalcoholic fatty liver disease spectrum reveals gene signatures for steatohepatitis and fibrosis. Sci Transl Med. (2020) 12:eaba4448. doi: 10.1126/scitranslmed.aba4448, PMID: 33268509

[38] Zhao J Zhang S Liu Y He X Qu M Xu G . Single-cell RNA sequencing reveals the heterogeneity of liver-resident immune cells in human. Cell Discov. (2020) 6:22. doi: 10.1038/s41421-020-0157-z, PMID: 32351704 PMC7186229

[39] Li H Qu L Yang Y Zhang H Li X Zhang X . Single-cell transcriptomic architecture unraveling the complexity of tumor heterogeneity in distal cholangiocarcinoma. Cell Mol Gastroenterol Hepatol. (2022) 13:1592–1609.e9. doi: 10.1016/j.jcmgh.2022.02.014, PMID: 35219893 PMC9043309

[40] Wu Y Yang S Ma J Chen Z Song G Rao D . Spatiotemporal immune landscape of colorectal cancer liver metastasis at single-cell level. Cancer Discov. (2022) 12:134–53. doi: 10.1158/2159-8290.CD-21-0316, PMID: 34417225

[41] Montironi C Castet F Haber PK Pinyol R Torres-Martin M Torrens L . Inflamed and non-inflamed classes of HCC: a revised immunogenomic classification. Gut. (2023) 72:129–40. doi: 10.1136/gutjnl-2021-325918, PMID: 35197323 PMC9395551

[42] Sun X He X Zhang Y Hosaka K Andersson P Wu J . Inflammatory cell-derived CXCL3 promotes pancreatic cancer metastasis through a novel myofibroblast-hijacked cancer escape mechanism. Gut. (2022) 71:129–47. doi: 10.1136/gutjnl-2020-322744, PMID: 33568427

[43] Zhou C Gao Y Ding P Wu T Ji G . The role of CXCL family members in different diseases. Cell Death Discov. (2023) 9:212. doi: 10.1038/s41420-023-01524-9, PMID: 37393391 PMC10314943

[44] Hoch T Schulz D Eling N Gómez JM Levesque MP Bodenmiller B . Multiplexed imaging mass cytometry of the chemokine milieus in melanoma characterizes features of the response to immunotherapy. Sci Immunol. (2022) 7:eabk1692. doi: 10.1126/sciimmunol.abk1692, PMID: 35363540

[45] Revel M Sautès-Fridman C Fridman W-H Roumenina LT . C1q+ macrophages: passengers or drivers of cancer progression. Trends Cancer. (2022) 8:517–26. doi: 10.1016/j.trecan.2022.02.006, PMID: 35288093

[46] Liu Y Zhang Q Xing B Luo N Gao R Yu K . Immune phenotypic linkage between colorectal cancer and liver metastasis. Cancer Cell. (2022) 40:424–437.e5. doi: 10.1016/j.ccell.2022.02.013, PMID: 35303421

[47] Son M . Understanding the contextual functions of C1q and LAIR-1 and their applications. Exp Mol Med. (2022) 54:567–72. doi: 10.1038/s12276-022-00774-4, PMID: 35562585 PMC9098383

[48] Zhou T Glaser SS . Pleiotropic effects of CD5L in hepatic inflammation and fibrosis. eBioMedicine. (2019) 44:22–3. doi: 10.1016/j.ebiom.2019.05.039, PMID: 31151931 PMC6604766

[49] Miyamoto Y Kikuta J Matsui T Hasegawa T Fujii K Okuzaki D . Periportal macrophages protect against commensal-driven liver inflammation. Nature. (2024) 629:901–9. doi: 10.1038/s41586-024-07372-6, PMID: 38658756

[50] Locati M Curtale G Mantovani A . Diversity, mechanisms, and significance of macrophage plasticity. Annu Rev Pathol Mech Dis. (2020) 15:123–47. doi: 10.1146/annurev-pathmechdis-012418-012718, PMID: 31530089 PMC7176483

[51] Anstee QM Reeves HL Kotsiliti E Govaere O Heikenwalder M . From NASH to HCC: current concepts and future challenges. Nat Rev Gastroenterol Hepatol. (2019) 16:411–28. doi: 10.1038/s41575-019-0145-7, PMID: 31028350

[52] Guilliams M Bonnardel J Haest B Vanderborght B Wagner C Remmerie A . Spatial proteogenomics reveals distinct and evolutionarily conserved hepatic macrophage niches. Cell. (2022) 185:379–396.e38. doi: 10.1016/j.cell.2021.12.018, PMID: 35021063 PMC8809252

[53] Roche PA Furuta K . The ins and outs of MHC class II-mediated antigen processing and presentation. Nat Rev Immunol. (2015) 15:203–16. doi: 10.1038/nri3818, PMID: 25720354 PMC6314495

[54] Cui X Liu H Shi T Zhao Q Li F Lv W . IFI27 integrates succinate and fatty acid oxidation to promote adipocyte thermogenic adaption. Adv Sci. (2023) 10:2301855. doi: 10.1002/advs.202301855, PMID: 37544897 PMC10558685

[55] Sharma A Seow JJW Dutertre C-A Pai R Blériot C Mishra A . Onco-fetal reprogramming of endothelial cells drives immunosuppressive macrophages in hepatocellular carcinoma. Cell. (2020) 183:377–394.e21. doi: 10.1016/j.cell.2020.08.040, PMID: 32976798

[56] Xu S Liu Y Ding Y Luo S Zheng X Wu X . The zinc finger transcription factor, KLF2, protects against COVID-19 associated endothelial dysfunction. Sig Transduct Target Ther. (2021) 6:266. doi: 10.1038/s41392-021-00690-5, PMID: 34253708 PMC8273371

[57] Zhang L Yu X Zheng L Zhang Y Li Y Fang Q . Lineage tracking reveals dynamic relationships of T cells in colorectal cancer. Nature. (2018) 564:268–72. doi: 10.1038/s41586-018-0694-x, PMID: 30479382

[58] Zhu Z Lou G Teng X-L Wang H Luo Y Shi W . FOXP1 and KLF2 reciprocally regulate checkpoints of stem-like to effector transition in CAR T cells. Nat Immunol. (2024) 25:117–28. doi: 10.1038/s41590-023-01685-w, PMID: 38012417 PMC10841689

[59] Sathe A Mason K Grimes SM Zhou Z Lau BT Bai X . Colorectal cancer metastases in the liver establish immunosuppressive spatial networking between tumor-associated *SPP1* + Macrophages and fibroblasts. Clin Cancer Res. (2023) 29:244–60. doi: 10.1158/1078-0432.CCR-22-2041, PMID: 36239989 PMC9811165

[60] Jaitin DA Adlung L Thaiss CA Weiner A Li B Descamps H . Lipid-associated macrophages control metabolic homeostasis in a trem2-dependent manner. Cell. (2019) 178:686–698.e14. doi: 10.1016/j.cell.2019.05.054, PMID: 31257031 PMC7068689

[61] Stankey CT Bourges C Haag LM Turner-Stokes T Piedade AP Palmer-Jones C . A disease-associated gene desert directs macrophage inflammation through ETS2. Nature. (2024) 630:447–56. doi: 10.1038/s41586-024-07501-1, PMID: 38839969 PMC11168933

[62] Geng F Chen J Song B Tang Z Li X Zhang S . Chaperone- and PTM-mediated activation of IRF1 tames radiation-induced cell death and the inflammatory response. Cell Mol Immunol. (2024) 21:856–72. doi: 10.1038/s41423-024-01185-3, PMID: 38849539 PMC11291999

[63] Trehan R Huang P Zhu XB Wang X Soliman M Strepay D . SPP1 + macrophages cause exhaustion of tumor-specific T cells in liver metastases. Nat Commun. (2025) 16:4242. doi: 10.1038/s41467-025-59529-0, PMID: 40335453 PMC12059142

[64] Lee YJ Jameson SC Hogquist KA . Alternative memory in the CD8 T cell lineage. Trends Immunol. (2011) 32:50–6. doi: 10.1016/j.it.2010.12.004, PMID: 21288770 PMC3039080

