# Supplementary material for: ScRNA-seq reveals dynamic macrophage heterogeneity in chronic liver disease progression and prognostic biomarkers KLF2/SPP1 in HCC
Source: Front Immunol. 2026 Feb 18;17:1766301. doi: 10.3389/fimmu.2026.1766301 (PMC12957215; doi:10.3389/fimmu.2026.1766301)
Supplement: Supplementary file 1 [file DataSheet1.docx]

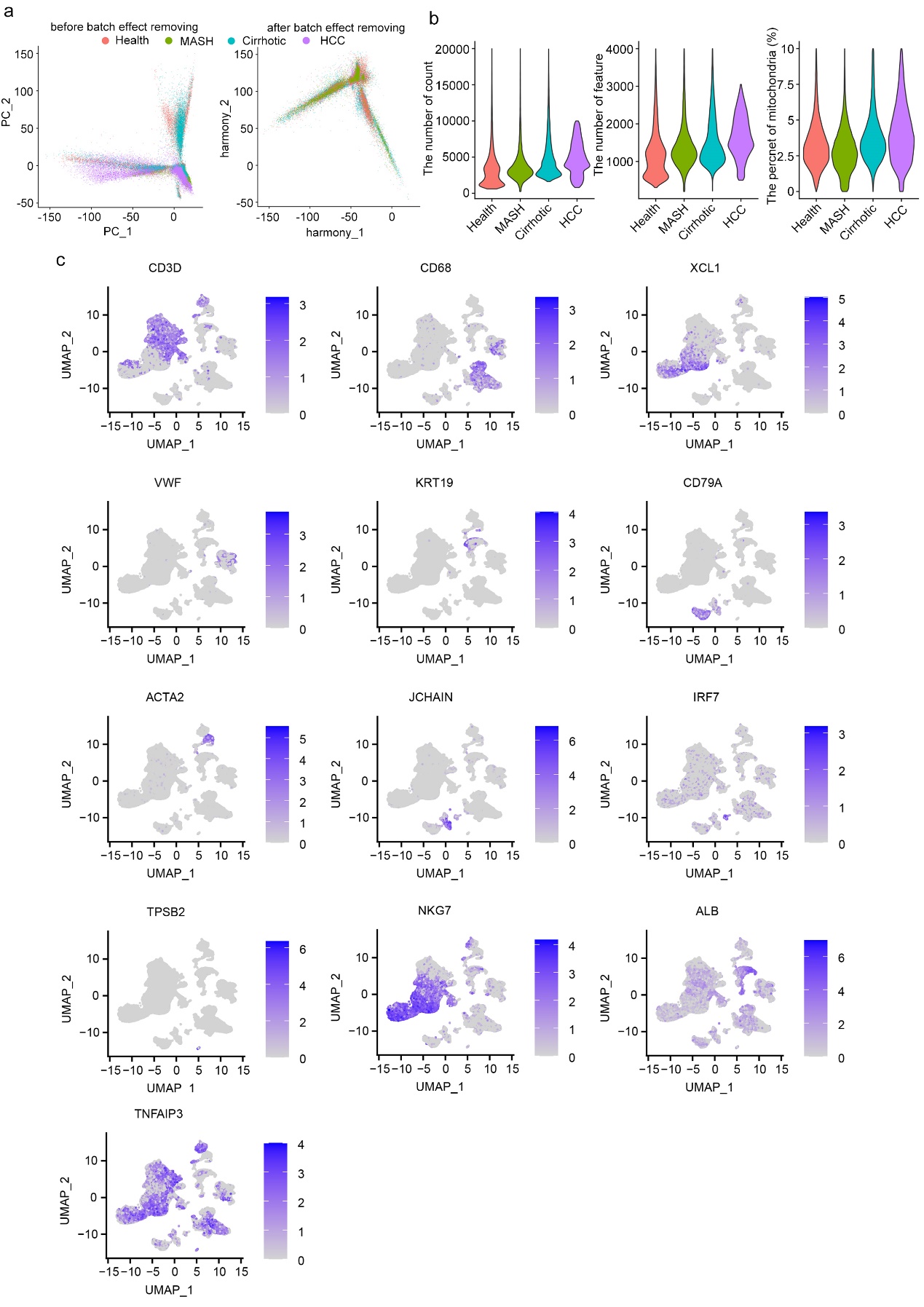


**Supplementary Fig. 1. Quality control of scRNA-seq data and the marker genes expression for cell type annotation.** a. The umap plot showing batch effect removing. b. The violin plots showing the number of genes expressed in the count matrix, the total counts per cell, and the percentage of mitochondrial genes. c. The umap plot showing the marker genes expression of main cell types.


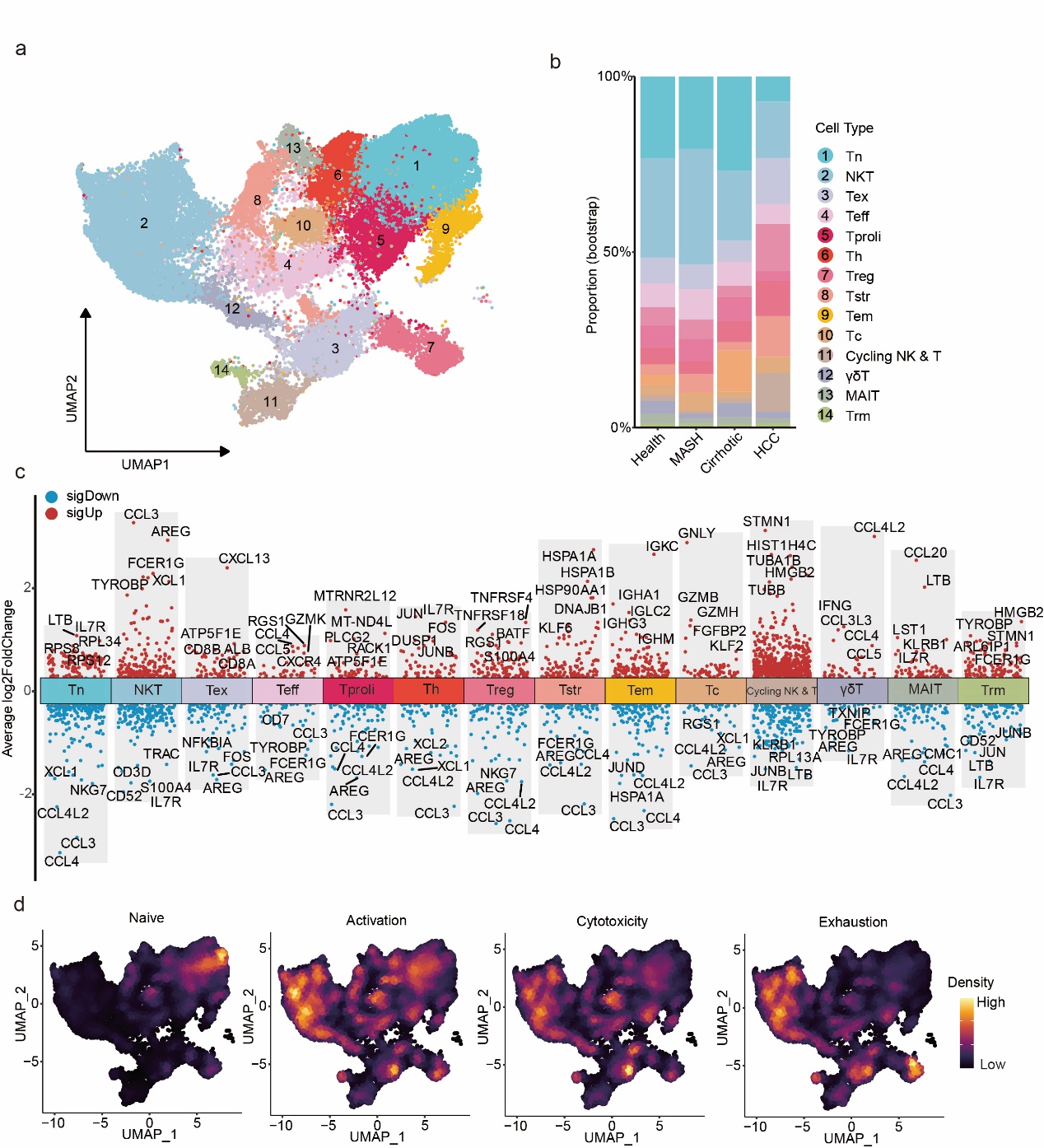


**Supplementary Fig. 2: Characterization of T cell subsets and states during CLDs.** a. UMAP plot showing the T cell and NKT clusters, colors represent different cell populations, dots represent individual cells. Tn, naïve T cell, NKT, natural killer T cell, Tex, exhausted T cell, Teff, effector T cell, Tproli, proliferating T cell, Th, T helper cell, Treg, T regulatory cell, Tstr, stress response T cell, Tem, effector memory T cell, Tc, cytotoxic T cell, Cycling NK & T, γδT, gamma delta T, MAIT, mucosal-associated invariant T cell and Trm, tissue-resident memory T cell. b. Histogram showing the proportion of T cell and NKT in different progression of CLDs. c. Differential gene expression analysis showing up- (red) and down- (blue) regulated genes of T cell subtypes. Adjusted p value < 0.05. d. Density plot showing naive, activation, cytotoxicity, and exhaustion properties of T cell subtypes.


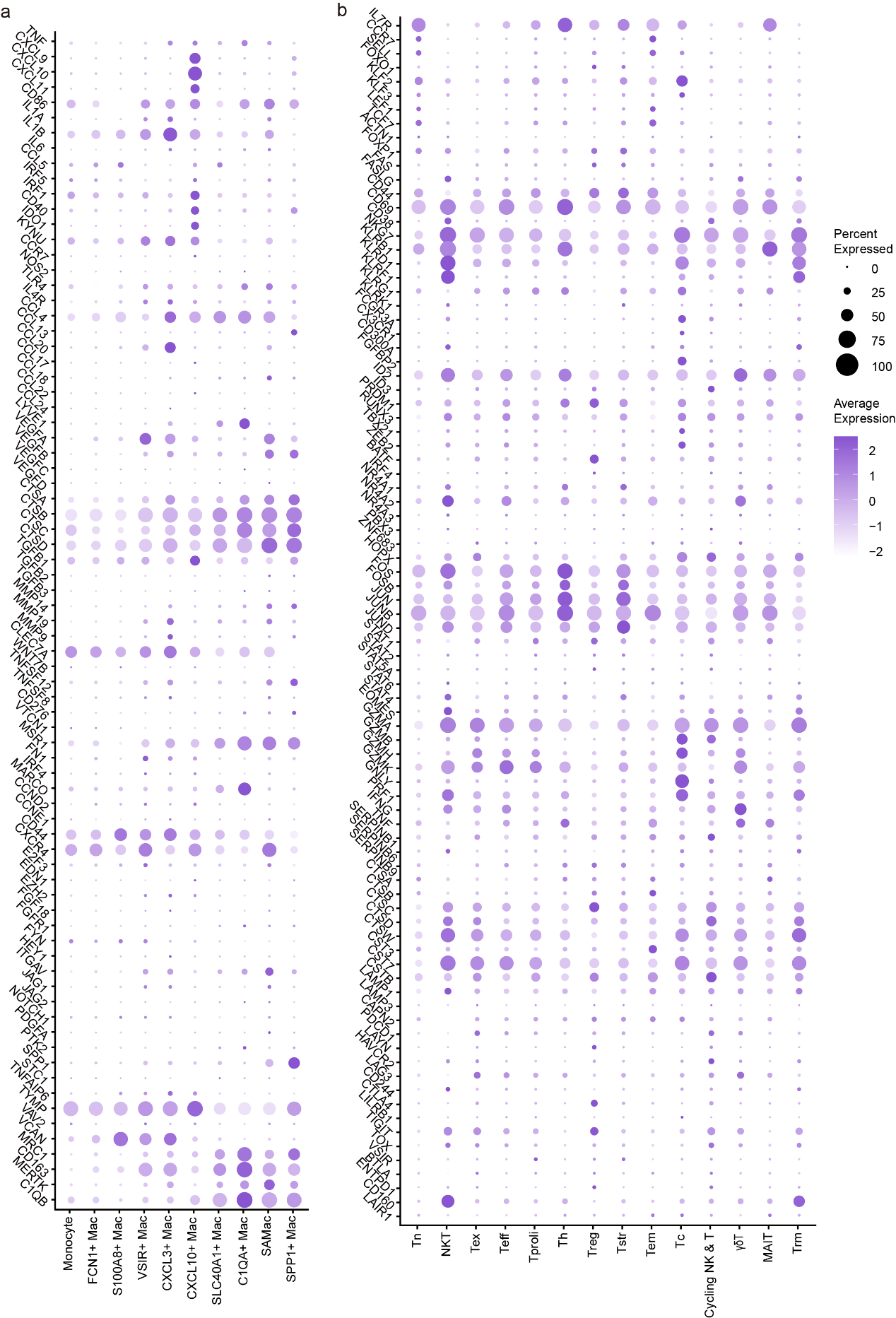


**Supplementary Fig. 3. Expression levels and frequencies of genes composing macrophage (a) and T cell (b) states.**


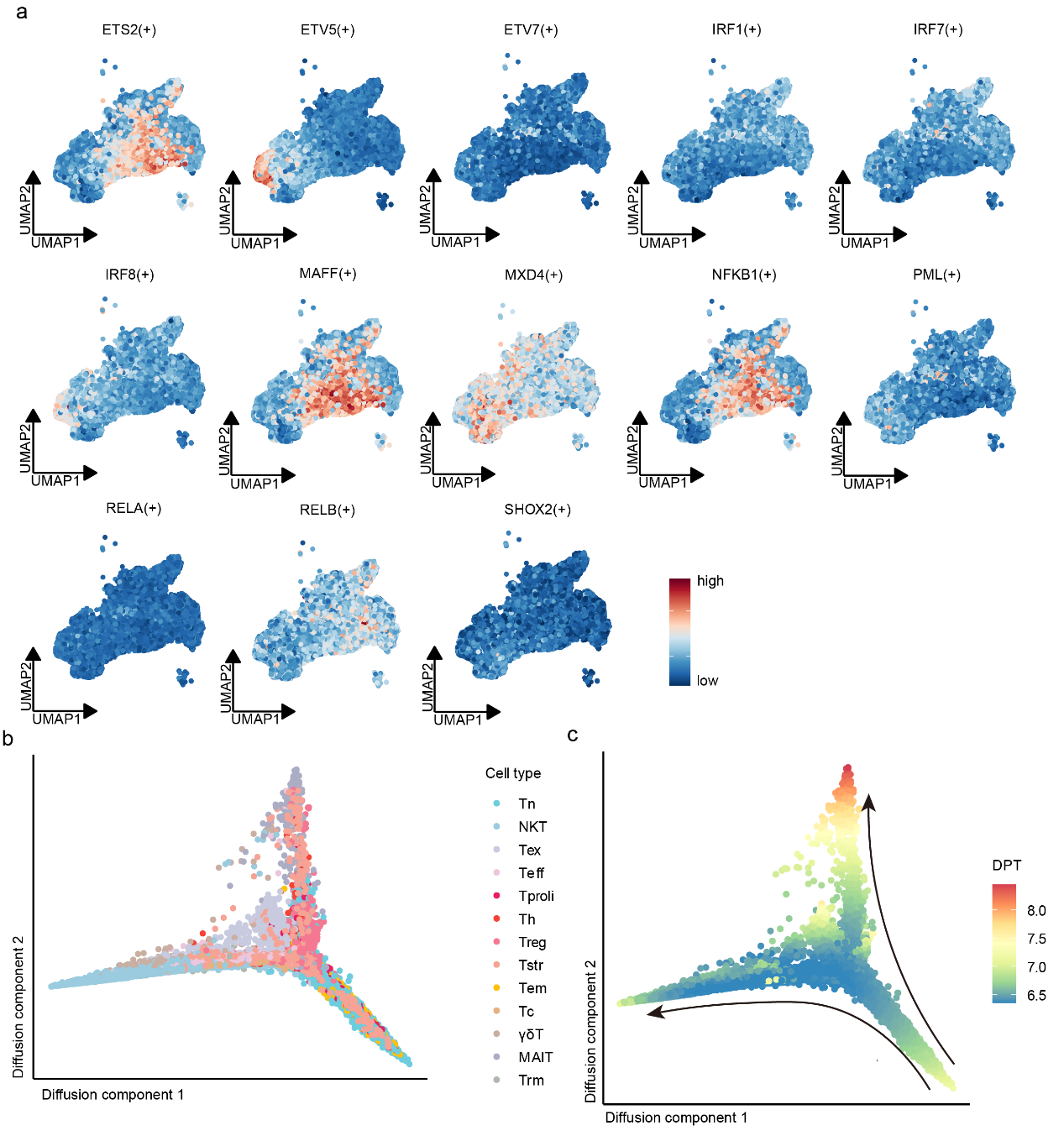


**Supplementary Fig. 4. Developmental trajectory and of transcription factor activity analysis during the progression of CLDs.** a. the specific transcriptional regulation score of macrophages. b. Pseudo-time trajectory projected of T cell subtypes.


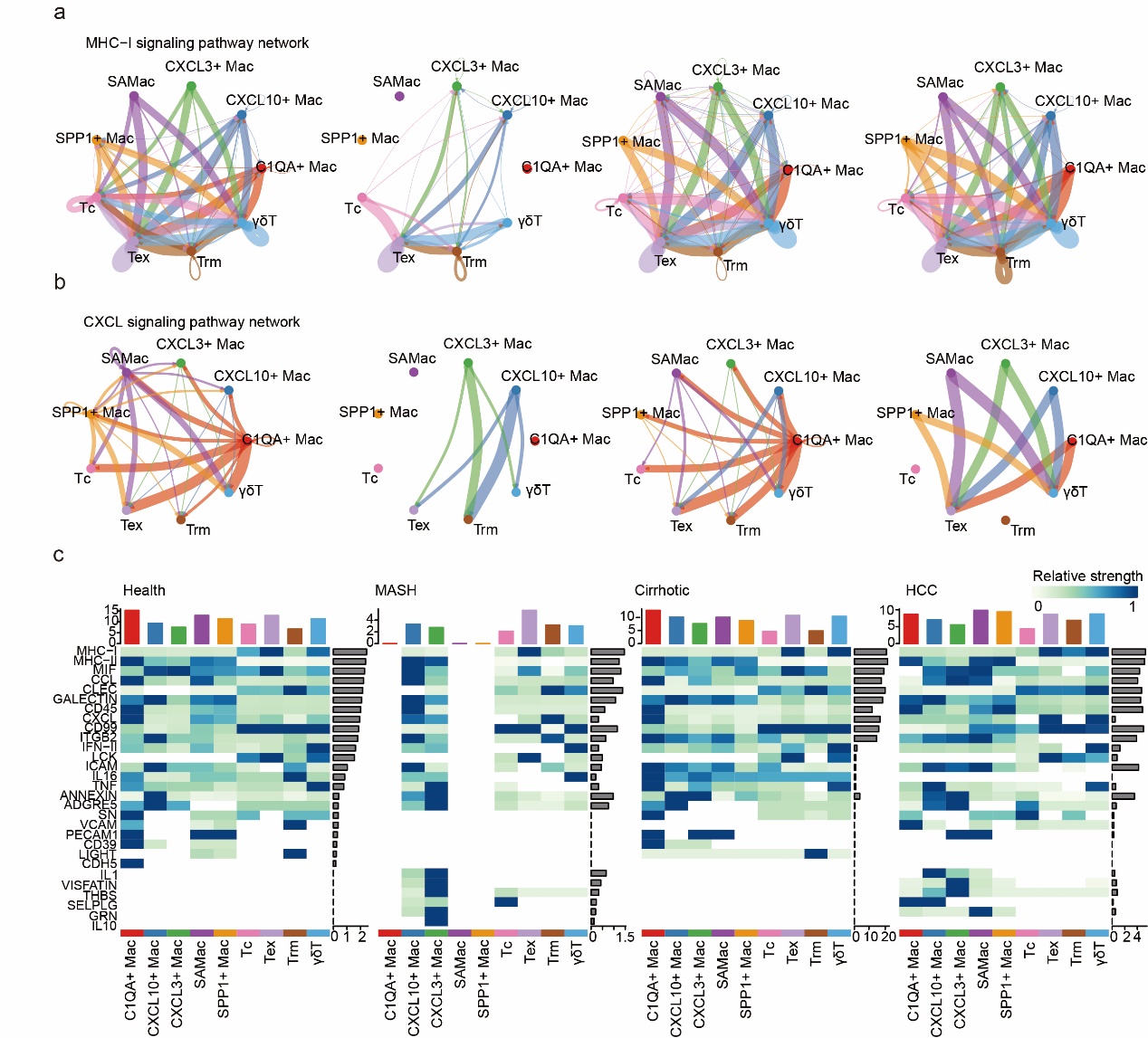


**Supplementary Fig. 5. The cell–cell interactions of macrophage and T cell subtypes in specific pathways during the progression of CLDs.** a-b. Circle plot showing the signaling pathway of MHC-I (a) and CXCL (b) pathway. c. The contribution of sigals for each cell types. The top colored bar plot showed the total signaling strength by summarizing all signaling pathways displayed in the heatmap. The right grey bar plot showed the total signaling strength by summarizing all cell types displayed in the heatmap.


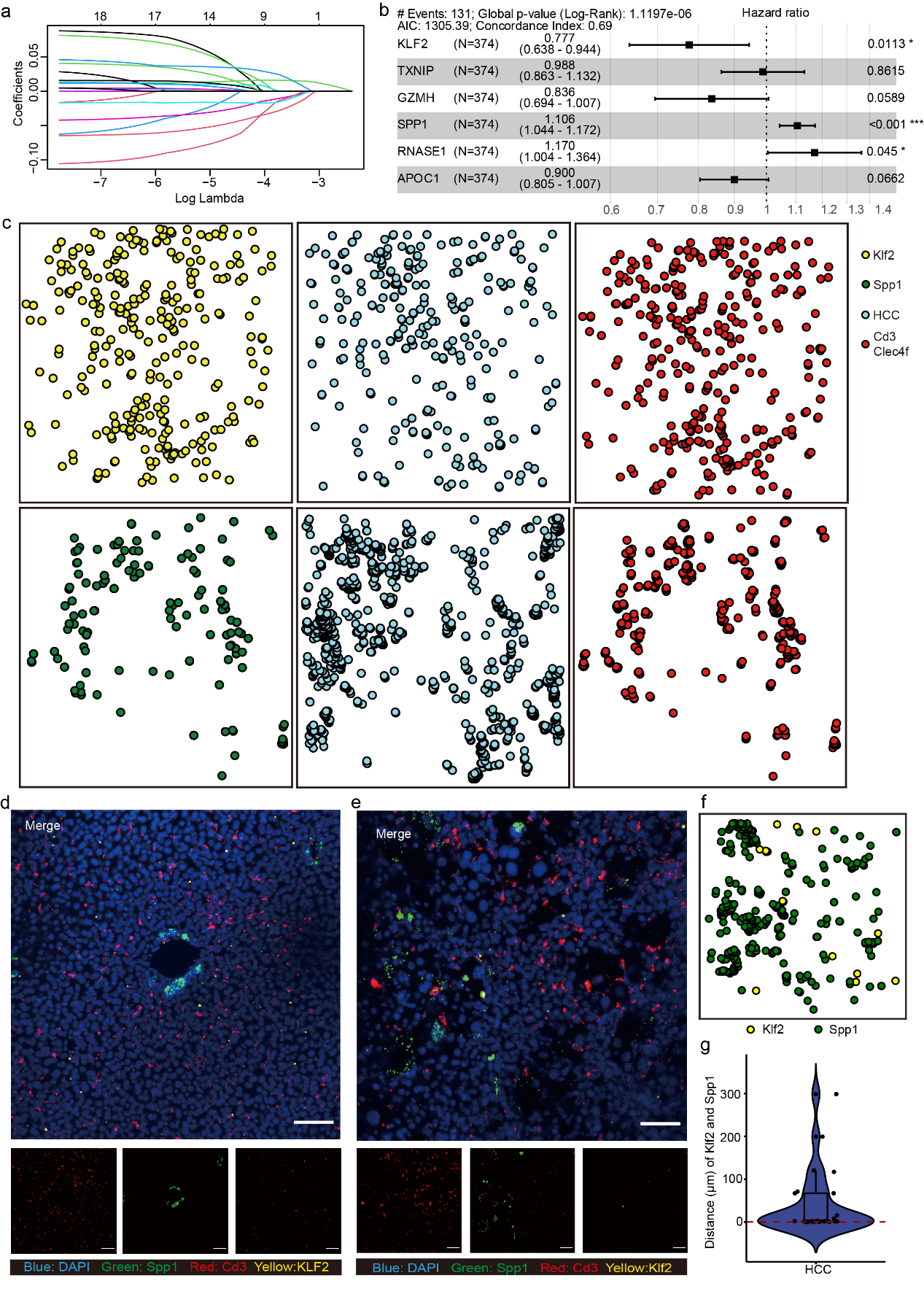


**Supplementary Fig. 6. Establishment and validation of prognostic signatures for HCC.** a-b. LASSO-Cox algorithms were combined to reveal prognostic signature for HCC. Univariate Cox regression analysis demonstrated that KLF2 (p = 0.013), SPP1 (p < 0.001) and RNASE1 (p = 0.045) were significant risk factor for overall survival of HCC.

d-e. Representative FISH micrographs (n ≥ 3) of Spp1 (green), Cd3 (red), Klf2 (yellow) and DAPI (blue) in healthy liver (e) and HCC (f). Scale bars: 200 µm. f. Scatterplots showed the distribution of Klf2 (Tc) and Spp1 (SPP1^+^ Mac) corresponding to FISH micrographs of Supplementary Fig. 6e. g. Violin plots showed the closest distance quantification of Tc and SPP1^+^ Mac corresponding cells identified on FISH micrographs of Supplementary Fig. 6e.

**Supplementary Table 1: The detail information of scRNA-seq data in this study**

| Accession number | Sample ID | Sample name | Disease | Gender | age |
| --- | --- | --- | --- | --- | --- |
| GSE136103 | GSM4041150 | Healthy1_Cd45+ | Health | Male | 57.4±7.9 |
| GSE136103 | GSM4041151 | Healthy1_Cd45-A | Health | Male |  |
| GSE136103 | GSM4041152 | Healthy1_Cd45-B | Health | Male |  |
| GSE136103 | GSM4041153 | Healthy2_Cd45+ | Health | Male |  |
| GSE136103 | GSM4041154 | Healthy2_Cd45- | Health | Male |  |
| GSE136103 | GSM4041155 | Healthy3_Cd45+ | Health | Male |  |
| GSE136103 | GSM4041156 | Healthy3_Cd45-A | Health | Male |  |
| GSE136103 | GSM4041157 | Healthy3_Cd45-B | Health | Male |  |
| GSE136103 | GSM4041158 | Healthy4_Cd45+ | Health | Female |  |
| GSE136103 | GSM4041159 | Healthy4_Cd45- | Health | Female |  |
| GSE136103 | GSM4041160 | Healthy5_Cd45+ | Health | Male |  |
| GSE159977 | GSM4851988 | PT-8 | MASH | NA | 52.6±14.7 |
| GSE159977 | GSM4851994 | PT-19 | MASH | NA |  |
| GSE159977 | GSM4851996 | PT-21 | MASH | NA |  |
| GSE136103 | GSM4041161 | Cirrhotic1_Cd45+ | Cirrhotic | Female | 56.6±5.8 |
| GSE136103 | GSM4041162 | Cirrhotic1_Cd45-A | Cirrhotic | Female |  |
| GSE136103 | GSM4041163 | Cirrhotic1_Cd45-B | Cirrhotic | Female |  |
| GSE136103 | GSM4041164 | Cirrhotic2_Cd45+ | Cirrhotic | Male |  |
| GSE136103 | GSM4041165 | Cirrhotic2_CD45- | Cirrhotic | Male |  |
| GSE136103 | GSM4041166 | Cirrhotic3_CD45+ | Cirrhotic | Male |  |
| GSE136103 | GSM4041167 | Cirrhotic3_Cd45- | Cirrhotic | Male |  |
| GSE136103 | GSM4041168 | Cirrhotic4_Cd45+ | Cirrhotic | Male |  |
| GSE136103 | GSM4041169 | Cirrhotic5_Cd45+ | Cirrhotic | Female |  |
| skrx2fz79n | NA | P1 | HCC | Male | 53 |
| skrx2fz79n | NA | P2 | HCC | Female | 49 |
| skrx2fz79n | NA | P3 | HCC | Male | 46 |
| skrx2fz79n | NA | P4 | HCC | Female | 70 |
| skrx2fz79n | NA | P5 | HCC | Male | 68 |
| skrx2fz79n | NA | P6 | HCC | Male | 48 |

**Supplementary Tables 2: signature gene sets relate to macrophage states**

| Macrophage states | Signature gene name |
| --- | --- |
| M1 | NOS2, TLR4, IL23, TNF, CXCL9, CXCL10, CXCL11, CD86, IL1A, IL1B, IL6, CCL5, IRF5, IRF1, CD40, IDO1, KYNU, CCR7 |
| M2 | IL4R, CCL4, CCL13, CCL20, CCL17, CCL18, CCL22, CCL24, LYVE1, VEGFA, VEGFB, VEGFC, VEGFD, EGF, CTSA, CTSB, CTSC, CTSD, TGFB1, TGFB2, TGFB3, MARCO, MMP14, MMP19, MMP9, CLEC7A, WNT7B, FASL, TNFSF12, TNFSF8, CD276, VTCN1, MSR1, FN1, IRF4 |
| Angiogenesis | CCND2, CCNE1, CD44, CXCR4, E2F3, EDN1, EZH2, FGF18, FGFR1, FYN, HEY1, ITGAV, JAG1, JAG2, MMP9, NOTCH1, PDGFA, PTK2, SPP1, STC1, TNFAIP6, TYMP, VAV2, VCAN, VEGFA |
| Phagocytosis | MRC1, CD163, MERTK, C1QB |

**Supplementary Tables 3: signature gene sets relate to T cell states**

| T cell states | Signature gene name |
| --- | --- |
| Naive | IL7R, CCR7, SELL, FOXP1, KLF2, KLF3, LEF1, TCF7, ACTN1, BTG1, BTG2, TOB1, FOXO1 |
| Activation | FAS, CD44, CD69, CD38, NKG7, KLRB1, KLRD1, KLRG1, CX3CR1, CD300A, FGFBP2, ID2, ID3, PRDM1, RUNX3, TBX21, ZEB2, BATF, NR4A1, NR4A2, HOPX, FOS, FOSB, FOSL2, JUN, JUNB, JUND, STAT1, STAT3, EOMES, AHR, FASLG, KLRF1, KLRK1, FCGR3A, IRF4, NR4A3, PBX3, ZNF683, STAT2, STAT5A, STAT6, STAT4 |
| Exhaustion | PDCD1, LAYN, HAVCR2, LAG3, CTLA4, TIGIT, TOX, VSIR, BTLA, ENTPD1, CD244, LILRB1, CD160, LAIR1 |
| Cytotoxicity | GZMA, GZMB, GZMH, GZMK, GZMM, GNLY, PRF1, IFNG, TNF, SERPINB9, CTSA, CTSB, CTSC, CTSD, CTSH, CTSW, CST7, CAPN2, PLEK, SERPINB1, SERPINB6, CST3, CSTB, LAMP1, LAMP3 |

**Supplementary Table 4: List of genes for fluorescence in situ hybridization in this study**

| Gene Name | Target sequences |
| --- | --- |
| C1qa | TGGAGACCTCTCAGGGATGGCTGGTGGCCTGTGTGCTGACCATGACCCTAGTATGGA  TCTCAGCCATTCGGCAGAACCCAATGACGCTTGGCAACGTGGTTATCTTTGACAAGGT  TGGATCGAAAAGGACCCCGCAAAGGGTCGCATTTACCAGGGCACTGAAGCCGACAGCA |
| Cd3 | TGTGCCTCAGCCTCCTAGCTGTTGGCACTTGCCAGGACGATGCCGAGAACATTGAA  ACGTACTTGTACCTGAAAGCTCGAGTGTGTGAGTACTGTGTGGAGGTGGACCTGACA  CATCGCCTTCTGTGGACCCAGATCCAGCCCTCCGAGCACCCTGCTACTCCTTGTTCT |
| Clec4f | ATGTTCTTTTTCAGTCGTTTTTCATCCACGTGATCTGAGAAAAATATTCTTAGTAGTGTT  AATGGACAATGTTAGTTCTCTGGTGCAGTTGCTTGGGAGCCATCTAGAGGATGTGAATGC  ATTCCTGGTACAGACTACAAGTTCTGGGGACCATTGGATTGGGCTCACTGACCAGGGCA |
| Cxcl3 | CACCAACCACCAGGCTACAGGGGCTGTTGTGGCCAGTGAGCTGCGCTGTCAGTGCC  ATGGTCAAGAAGTTTGCCTCAACCCCCAAGGCCCCAGGCTTCAGATAATCATCAAGAAGA |
| Cxcl10 | TGCCGTCATTTTCTGCCTCATCCTGCTGGGTCTGAGTGGGACTCAAGGGATCCCTCTC  GATGAGCAGAGATGTCTGAATCCGGAATCTAAGACCATCAAGAATTTAATGAAAGCGTTT |
| Klf2 | CTCAGCGAGCCTATCTTGCCGTCCTTTGCCACTTTCGCCAGCCCGTGCGAGCGCGG  AAAATCCTCCCGAGCCCCCGCCGCAGCCCCCGCCGCCTGCCTTCTACTACCCGGA  AACCCGTTCCCGCCGCCCTTCGGTCCCGGCCCCAGCTTCGGCGGTCCCGGCCCCG |
| Spp1 | AAAGGATGACTTTAAGCAAGAAACTCTTCCAAGCAATTCCAATGAAAGCCATGACCAC  AAAGTCTAGGAGTTTCCAGGTTTCTGATGAACAGTATCCTGATGCCACAGATGAGGACCT  AAGTTTCACAGCCACAAGGACAAGCTAGTCCTAGACCCTAAGAGTAAGGAAGATGATA |
| Trem2 | ACCTCTCCACCAGTTTCTCCTGCTGCTGATCACAGCCCTGTCCCAAGCCCTCAACACCAC  GGTCCTGCAGAAAGTACTGGTGGAGGTGCTGGAGGACCCTCTAGATGACCAAGATGCTGGAG  GGGAACACCTGTGGTCAGAGGGCTGGACTGTGGCCAAGATGCTGGGCACCAACTTCAGAT |
| Akt | GAAACGCCTGAGGAGCGGGAAGAATGGGCCACCGCCATTCAGACTGTGGCAGATGGA  CATGTACGAGATGATGTGTGGCCGCCTGCCCTTCTACAACCAGGACCACGAGAAGCTG |
| Nras | AATCCAGCTAATCCAGAACCACTTTGTAGATGAATATGATCCCACCATAGAGGATTCTTACAG  TCATTGAAACCTCAGCCAAGACCAGACAGGGTGTTGAAGATGCTTTTTACACACTGGTA |
